# Occurrence, Epidemiological Aspects and Multilocus Sequence Typing of Pear Decline Phytoplasma in Southern Italy

**DOI:** 10.3390/plants15213432

**Published:** 2026-11-04

**Authors:** Carmine Marcone, Carmine Palmieri, Alberto Sellitto, Simone Russo, Donatella Battaglia, Matilde Tessitori, Andrea Luvisi, Rosemarie Tedeschi

**Affiliations:** 1 Department of Pharmacy, University of Salerno, 84084 Fisciano, Italy; carminepalmieri97@gmail.com (C.P.); a.sellitto19@studenti.unisa.it (A.S.); s.russo129@studenti.unisa.it (S.R.); 2 Department of Agricultural, Forestry, Food and Environmental Sciences (DAFE), University of Basilicata, 85100 Potenza, Italy; donatella.battaglia@unibas.it; 3 Department of Agriculture, Food and Environment (Di3A), University of Catania, 95123 Catania, Italy; mtessitori@unict.it; 4 Department of Biological and Environmental Sciences and Technologies, University of Salento, 73100 Lecce, Italy; andrea.luvisi@unisalento.it; 5 Department of Agricultural, Forest, and Food Sciences (DISAFA), University of Turin, 10095 Grugliasco, Italy; rosemarie.tedeschi@unito.it

**Keywords:** fruit trees, ‘*Candidatus* Phytoplasma pyri’, apple proliferation group, decline symptoms, pear psylla, PCR assays, phylogenetic analysis, rDNA, *aceF* genotypes, genetic variability

## Abstract

Visual symptoms assessment and PCR assays using rDNA-directed primers, followed by RFLP analysis, were employed to update and extend knowledge on the occurrence of pear decline (PD) phytoplasma in several pear-growing areas of the neighboring regions Campania, Basilicata and Calabria of southern Italy, including those which had previously not been examined. The PD phytoplasma was detected in all symptomatic pear trees examined and in 46 out 63 pools of the pear psylla *Cacopsylla pyri* insects captured in the surveyed pear orchards. Disease incidence of PD ranged from 10 to 65% in the pear orchards examined. Severe PD symptoms were observed in symptomatic pear trees grafted onto BA29 quince rootstock. Multilocus sequence typing (MLST) analysis of *aceF, pnp, imp* and *secY* gene sequences revealed a considerable genetic variability among 30 selected PD phytoplasma-infected samples examined in southern Italy. The highest number of genotypes was observed for *pnp* and *secY* genes (five genotypes for each), whereas the highest sequence dissimilarity value between the samples under study 15.1 occurred in the *aceF* gene. For each gene examined, the most frequently identified genotypes were A24, P4, I40 and S13, respectively. The detected multilocus profiles are compatible with the hypothesis of possible interspecies recombination between ‘*Ca*. Phytoplasma pyri’ and ‘*Ca.* Phytoplasma prunorum’, although alternative explanations including mixed infections, preferential amplification and PCR-mediated recombination are not excluded. Fifteen distinct multilocus profiles were obtained from the selected samples and compared with previously published profiles using a similarity network. These findings extend knowledge on the multilocus diversity of PD phytoplasma in southern Italy.

## 1. Introduction

Pear decline (PD) is the most serious vector-borne and graft-transmissible disease of the cultivated European or French pear (*Pyrus communis*), causing significant crop losses as well as stunting and dying of trees grown on susceptible rootstocks [1]. This disease is widespread in all pear-growing areas of North America and Europe [2]. It was first reported in British Columbia in 1948 [3] and only a few years later spread along the Pacific Coast appearing in Washington, Oregon and California [4,5,6]. In ensuing years, PD was reported in eastern North America [1]. There is indication that PD was present in Italy long before it was found in North America. A decline-like disorder of pear called “pear moria” has been observed in northern Italy since at least 1908 [7,8,9,10,11]. Joint studies carried out by both American and Italian researchers in the summer of 1961 showed that the moria disease of pear occurring in Italy was the same as pear decline in North America [12,13,14]. Therefore, it appears that PD is not of North American but rather of European origin and was introduced to the Americas with *P. communis* cultivars and rootstocks from Europe [1]. The disease can be extremely catastrophic. It killed more than one million pear trees in California between 1959 and 1962 and over 63,000 pear trees in Trentino-Alto Adige of northern Italy between 1944 and 1947 [14,15]. Over the last years, PD has also been recorded in Taiwan, Azerbaijan, Lebanon, Iran, Turkey, Belarus, Chile, Argentina, Jordan and Israel [16,17,18,19,20,21,22,23,24,25,26,27]. Decline-like disorders of pear have been observed in several other countries and regions, but their relationship to PD has not been established [1].

Phytoplasmas are obligate, intracellular bacteria of the class *Mollicutes* that are associated with diseases of a wide range of plant species worldwide, including temperate fruit trees, and in some cases with the insects that act as vectors [28,29,30]. These wall-less prokaryotes colonize the plants systemically by inhabiting the phloem sieve tube elements, which are the only plant cells commonly invaded, and are transmitted in nature from plant to plant by phloem-feeding homopteran insects [31,32]. Phytoplasmas are not sap transmissible. However, they can be transmitted inadvertently in nurseries during propagation of plant material by grafting. Also, several phytoplasmas have been transmitted to experimental hosts through dodder (*Cuscuta* spp.) bridges [33,34]. The main effect of phytoplasma infections is the impairment of phloem functions, which may account for the symptoms observed on affected plants [28,35]. Because of the inability to culture phytoplasmas under axenic conditions, their taxonomic classification is inevitably based on phylogenetic traits. Currently, phytoplasmas are assigned to the provisional genus ‘*Candidatus* Phytoplasma’ of the class *Mollicutes* based primarily on 16S rDNA sequence analysis [36]. This genus comprises 39 taxonomic groups (or 16Sr groups) and several subgroups as well as 58 formally described ‘*Candidatus* Phytoplasma’ species [36,37,38,39,40]. Sequencing of multiple PCR products or multilocus sequence typing (MLST) analysis of a number of housekeeping genes seems to be one of the most promising tools for finer strain differentiation and to deeply investigate genetic variability and phylogenetic relationships among phytoplasmas. This analysis can be used to provide supporting data for assigning species rank to phytoplasmas when 16S rDNA sequence differences are below the threshold value of 1.35% [37,38].

PD is caused by ‘*Ca*. Phytoplasma pyri’, a member of the apple proliferation (AP) phytoplasma group or 16SrX group, subgroup 16SrX-C [1,41]. This pathogen is phylogenetically closely related to other AP-group members, notably ‘*Ca*. Phytoplasma mali’ and ‘*Ca*. Phytoplasma prunorum’, the causative agents of AP and European stone fruit yellows (ESFY) diseases, respectively, as well as to the peach yellow leaf roll (PYLR) phytoplasma and the phytoplasma identified in decline-affected Japanese pear (*P. pyricola*) in Taiwan (PDTW) [1,29,41]. In most studies in which ribosomal and non-ribosomal DNA sequences were employed, ‘*Ca*. Phytoplasma pyri’ proved to be indistinguishable from the PYLR agent [41,42,43]. Differences between the two phytoplasmas were observed in the *imp* gene [44,45]. The PYLR phytoplasma is regarded as a subtype of ‘*Ca*. Phytoplasma pyri’ and is also designated as PD/PYLR phytoplasma [41,42,43]. ‘*Ca*. Phytoplasma pyri’ differs from the PDTW agent in the 16S rDNA sequences by the same order of magnitude as with ‘*Ca*. Phytoplasma mali’ and ‘*Ca*. Phytoplasma prunorum’. Thus, the PDTW agent, which is known to occur only in Taiwan, represents a distinct taxonomic entity [1,16].

Similarly to other AP-group fruit tree phytoplasmas, ‘*Ca*. Phytoplasma pyri’ is a homogeneous pathogen at ribosomal DNA (rDNA) sequence level. A considerable genetic diversity within this pathogen has been observed in less-conserved, non-ribosomal genes including *aceF*, *pnp, imp* and *secY* [18,46,47,48,49,50]. In particular, it has been shown through MLST analysis of the mentioned genes that some Spanish, Azerbaijanenese and Italian isolates of ‘*Ca*. Phytoplasma pyri’ possess some genes of ‘*Ca.* Phytoplasma prunorum’, thus supporting the existence of interspecies recombination between these two taxa [18,48,51,52,53]. ‘*Ca*. Phytoplasma pyri’ has been observed in naturally infected trees of *P. communis*, *P. pyricola*, *P. ussuriensis*, *P. calleryana*, *P. elaeagrifolia* and quince (*Cydonia oblonga*). This pathogen has been transmitted by grafting to a large number of *Pyrus* species. Therefore, it is likely that all *Pyrus* species are hosts of PD agent [1]. In addition, PD phytoplasma has been detected through PCR-based methods in naturally infected trees of European hazel (*Corylus avellana*), peach (*Prunus persica*), nectarine (*P. persica* var. *nectarina*), almond (*P. dulcis*) and sweet cherry (*Prunus avium*) as well as in naturally infected hawthorn (*Crataegus monogyna*) plants [1,54,55,56,57]. By psylla feeding, ‘*Ca.* Phytoplasma pyri’ has been transmitted from diseased pear trees to periwinkle (*Catharanthus roseus*) [58] and via dodder (*C*. *odorata*) bridges to periwinkle and subsequently to tobacco (*Nicotiana occidentalis* and *N. tabacum*) [59]. Like other AP-group fruit tree phytoplasmas, ‘*Ca.* Phytoplasma pyri’ is transmitted naturally by *Cacopsylla* species (for review, see [60]). *C. pyricola* is vectoring the pathogen in North America, whereas in Europe, the reported vectors are *C. pyricola*, *C. pyri*, *C. pyrisuga* and *C. bidens*. Of these, *C. pyri* has been identified as the main vector in some southern European countries including Italy [61,62,63]. *C. bidens* is known as a vector of PD agent also in Jordan and Israel [25,27,57,64]. PDTW phytoplasma infections were detected in *C. qianli* and *C. chinensis* collected in Japanese pear orchards in Taiwan. For the latter species, the vectorship has also been confirmed through transmission experiments [16,65]. The expression of symptoms on PD-affected pear trees largely depends on the rootstock and stage of the disease. Generally, three different syndromes can be distinguished. These are quick decline, slow decline, and reddening of the foliage, which is often associated with leaf curl [1].

PD disease has been reported in the neighboring Campania, Basilicata, Calabria and Apulia regions of southern Italy on the basis of symptom evaluation and PCR assays using group-specific ribosomal primers and RFLP analysis of PCR-amplified 16S rDNA sequences [66,67,68,69,70]. Also, a few isolates of the PD phytoplasma recorded in PD-affected pear trees in Basilicata during autumn 1997 were examined through sequence and phylogenetic analysis of either 16S rDNA gene (one isolate) [71] or 16S rDNA and various less-conserved genes (two isolates) [50]. No information is available on relationships of PD phytoplasma to insect vector occurring in southern Italy. Thus, the aim of the present work was to update and extend knowledge on the occurrence of PD in selected pear-growing areas of southern Italy through visual symptoms assessment and PCR assays, identify the pear psylla species collected in three affected orchards in Campania, assess the presence of PD phytoplasma DNA in these insects, and elucidate the genetic variability of the PD agent through MLST analysis.

## 2. Results

### 2.1. Symptomatology, Disease Incidence and Pear Psylla Collection

**Figure 1:**
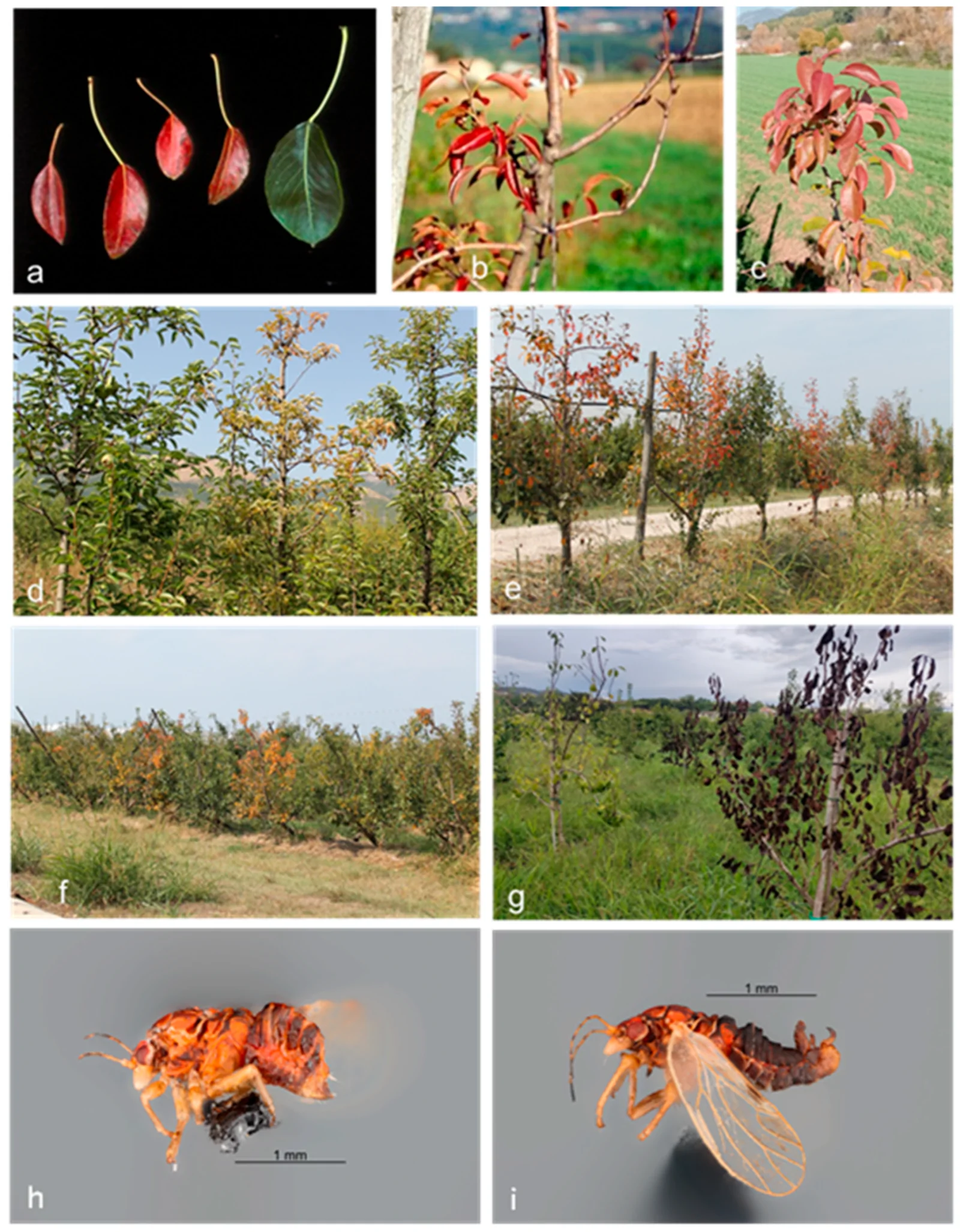
Symptoms of pear decline (PD) in cultivated European pear (*Pyrus communis*) and adults of *Cacopsylla pyri*. (**a**–**c**) Pear leaves and trees showing foliar reddening, reduced leaf size, sparse foliage and leaf curling; a healthy leaf is shown on the right in (**a**). (**d**) Pear tree showing reduced terminal growth, sparse foliage and yellowing leaves with slightly upturned margins; healthy-appearing trees are visible on either side. (**e**) Declining pear trees with premature leaf drop among healthy-appearing trees in the same row. (**f**) Pear trees showing premature yellowing and reddish discoloration at the edge of an orchard. (**g**) Slowly declining pear tree (left) and pear tree showing sudden wilting (right). (**h**) Adult female of *Cacopsylla pyri*. (**i**) Adult male of *C. pyri*.

The symptoms observed on diseased pear trees were similar to those previously described from other geographic areas and included mainly those typical of the slow decline and reddening of the foliage syndromes [1]. Affected trees showed no or reduced terminal growth. The leaves were few, small, light green and leathery with margins slightly up-rolled. Such leaves became abnormally red in late summer and dropped prematurely in autumn. Diseased trees were low in vigor and had erratic bloom, and fruit set and fruit size were impaired. Very often, foliar reddening was associated with leaf curling, a symptom characterized by downward curling of leaves from tip to midrib, while the margins were rolled upward along the longitudinal axis. Several severely affected pear trees died during the entire observation period. Also, some slow decline-diseased trees suddenly wilted and died in July and August of each year of observation in the presence of abnormally hot and dry weather conditions (Figure 1). The root system of severely affected trees was reduced in size in comparison to that of healthy trees. The number of affected trees in individual orchards ranged from 10% (198 symptomatic trees out of 1980 pear trees assessed) in the orchard located near Sibari (orchard Sib) to 65% (540 symptomatic trees out of 830 pear trees assessed) in the orchard located near Battipaglia (orchard Bat 1). High disease incidences were also recorded in the pear orchard located near Battipaglia (orchard Bat 2) and in the pear orchard near Pontecagnano Faiano (orchard Pont), which were 62% (1250 symptomatic trees out of 2015 pear trees assessed) and 58% (535 symptomatic trees out of 927 pear trees assessed), respectively. For the remaining orchards, disease incidence was 12% (507 symptomatic trees out of 4223 pear trees assessed) in the orchard located near Caserta (orchard Cas), 22% (586 symptomatic trees out of 2668 pear trees assessed) in the orchard located near Paterno (orchard Pat) and 18% (184 symptomatic trees out of 1020 pear trees assessed) in the orchard located near Grumento Nova (orchard Gru). Disease incidence was evaluated and monitored throughout the investigation period 2023–2025. Thus, the mentioned data of disease incidence were confirmed in the final assessment carried out at the end of the investigation period, which is September–October 2025.

A total of 640 pear psyllids were collected by means of yellow sticky traps during 2024–2025 in three pear orchards located in the Campania region near Battipaglia (orchard Bat 1 and Bat 2) and Pontecagnano Faiano (orchard Pont), which were the most severely affected (Figure S1). In particular, in 2024, 104, 98 and 153 psyllids were collected in orchards Bat 1, Bat 2 and Pont, respectively. In 2025, 84, 79 and 122 psyllids were collected in the same orchards, respectively. All captured psyllids were morphologically identified as *Cacopsylla pyri* (Figure 1h,i). No other *Cacopsylla* spp. were recorded. Although the total number of trap-days for each year was 3952, notably 1064, 1824 and 1064 for orchards Bat 1, Bat 2 and Pont, respectively, psyllids were consistently collected from September through mid-October of each year. Only a few or no psyllids were collected in spring and summer due either to insecticide treatments applied during these seasons for control of relevant pear pests including pear psylla, codling moth and aphids or to an abnormally hot summer.

### 2.2. PCR Analysis

A total of 181 symptomatic pear trees examined by PCR amplification using ribosomal DNA (rDNA) primer pairs P1/P7, fO1/rO1 and fPD/rO1 proved to be infected. However, 25 of these trees tested phytoplasma positive only through nested PCR assays in which amplification products obtained with primer pair P1/P7 were re-amplified with either AP group-specific primer pair fO1/rO1 or PD-slightly specific primer pair fPD/rO1. An amplification product of the expected size was also obtained from phytoplasma reference strains AT, PD1 and GSFY1 with primer pairs P1/P7 and fO1/rO1 and from strain PD1 with primer pair fPD/rO1. No amplification products were obtained from non-symptomatic pear trees and healthy pear seedlings (healthy plant controls). All pear trees that tested phytoplasma positive only through nested PCR were resampled and re-examined by PCR assays. As in the first examination, all trees tested positive only in nested PCR assays with the same primer combinations described above. Examples of the products obtained in nested PCR with primer pair fO1/rO1 are shown in Figure S2. In all phytoplasma-positive trees, the target DNA was amplified in samples from both stem and roots. A total of 630 pear psyllids, i.e., 63 pools each containing 10 individuals, were examined by PCR assays. Ten out of the 640 pear psyllids collected were not processed by molecular methods because of their advanced state of deterioration. Of the 63 pools examined, 46 (73.0%) tested phytoplasma positive using the ribosomal primer pairs described above. Five pools tested positive only by nested PCR. The percentage of positive pools does not represent individual infection prevalence. The estimated individual infection probability obtained from pooled testing under appropriate assumptions was 12% (95% exact confidence interval: 8.8–16.5%). Non-ribosomal DNA primer pairs employed in this study amplified the target DNA in all samples from diseased pear trees and pear psylla that tested phytoplasma positive with ribosomal DNA primer pairs. Also, samples from two pools of pear psylla examined by PCR assays using the primer pair 16SR21/16S22 gave an amplification product of the expected size. In none of the primer pairs employed in either direct or nested PCR assays was an amplification product obtained from reaction mixtures devoid of DNA template (negative controls) and extraction blank controls (Figure S2). Also, all samples from pear trees and psylla that proved to be phytoplasma negative tested positive when amplification inhibition controls were added.

### 2.3. RFLP Analysis of PCR Products

After digestion of PCR products obtained with the group-specific primer pair fO1/rO1 with SspI restriction endonuclease, DNA of the reference strain AT of the AP phytoplasma was cleaved due the presence of a unique restriction site in the 16S rDNA sequences. In contrast, the corresponding fragments obtained from diseased pear trees, pear psylla and reference strains PD1 and GSFY1 of PD and ESFY phytoplasmas, respectively, were not cleaved by this enzyme (Figure S3). When the same ribosomal fragments were digested with RsaI restriction endonuclease, all samples from diseased pear trees and pear psylla as well as reference strains PD1 and AT showed the same restriction profile. This profile was different from that of strain GSFY1, which showed an additional restriction site in the amplified sequences.

### 2.4. Sequencing of DNA and Data Analysis

Sequences of *aceF*, *pnp*, *imp* and *secY* genes were determined and compared for 30 PD phytoplasma-infected samples recorded in southern Italy, which were selected among those with the highest quality and intensive PCR products from each surveyed orchard. Of these, 24 samples originated from PD-infected pear trees, whereas six samples were obtained from pools of the pear psylla *C. pyri*. For each gene examined, the mentioned samples were compared to each other and to other PD phytoplasma strains and related phytoplasmas whose sequence is available in the GenBank database (Table S1).

Four different *aceF* genotypes that proved to be indistinguishable from those previously reported by Danet et al. [18] from various European countries including Italy as well as Azerbaijan and Turkey, namely A24, A10, A12 and A8, were identified among the selected PD phytoplasma-infected samples examined (Table 1). The A24 genotype was the most common among the selected samples under study and occurred in seven samples (PD2866, PD2881, PD2902, PD2973, PD2974, PD2977 and PD2994) from PD-affected pear trees and five samples (PD3204, PD3207, PD3211, PD3217 and PD3227) from PD phytoplasma-infected pear psylla (Table 1). A10 genotype was identified in nine samples (PD2639, PD2645, PD2882, PD2890, PD2896, PD2897, PD2906, PD2907 and PD2934), which were obtained from PD-affected pear trees and one sample (PD3223) from PD phytoplasma-infected pear psylla. The A12 genotype was identified in the sample PD3025, which was recorded in PD-affected pear trees. Genotype A8 was identified in seven samples (PD2642, PD2799, PD2800, PD2871, PD2886, PD3029 and PD3031), which were all from PD-affected pear trees. Unlike genotypes A24, A10 and A12, genotype A8 corresponded to *aceF* sequences of ‘*Ca*. Phytoplasma prunorum’ [18]. The presence of A8 genotype is one of the features of the corresponding multilocus profiles, which together are compatible with the hypothesis of possible recombination between *Ca*. Phytoplasma pyri’ and *‘Ca.* Phytoplasma prunorum’ as reported previously [18,48,51,52,53]. However, mixed infection, preferential amplification and PCR-mediated recombination are not excluded.

**Figure 2:**
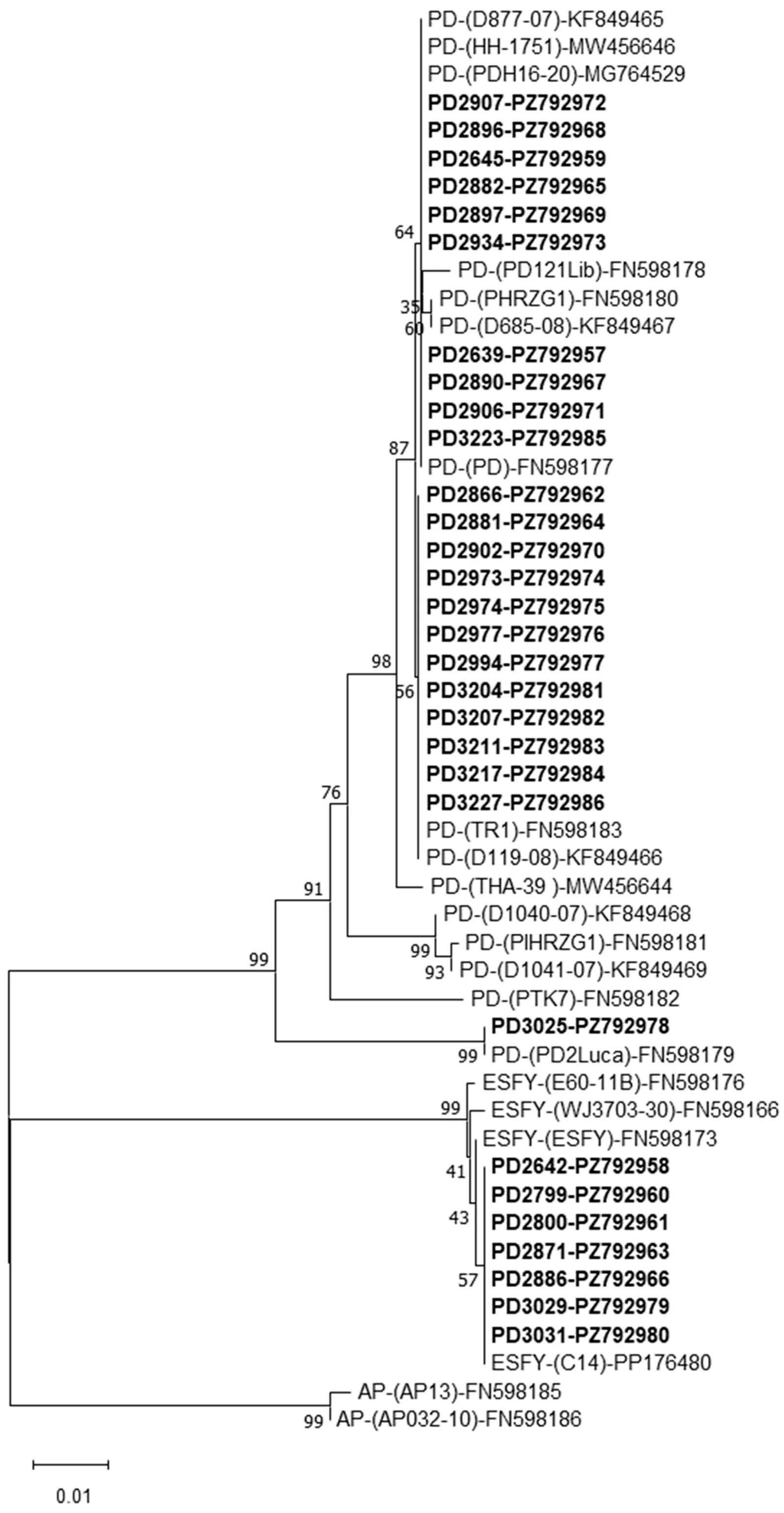
Phylogenetic tree constructed using the neighbor-joining method implemented in MEGA software, version 12 [73] with *aceF* gene sequences from PD phytoplasma-infected samples from southern Italy (in bold type) and related phytoplasma strains. The neighbor-joining tree analysis was conducted using the following parameters: substitution model, (p-distance); gap handling, pairwise deletion; alignment length, 757 bp; trimming criteria, manual trimming using MEGA’s alignment explorer tool. Bar represents a phylogenetic distance of 0.01 nucleotide substitutions per site. GenBank accession number is provided for each phytoplasma-positive sample/strain. Bootstrap values are shown on branches of the phylogenetic tree. See Table S1 for phytoplasma sample/strain abbreviations and further information about the related phytoplasma strains examined.

Genotypes corresponding to the *aceF* sequences of ‘*Ca*. Phytoplasma pyri’ showed *aceF* sequence similarity, which varied from 94.6 (A12 versus A24) to 99.9% (A10 versus A24). These genotypes shared 84.9–85.8% *aceF* gene sequence similarity with A8 genotype corresponding to the *aceF* sequences of ‘*Ca*. Phytoplasma prunorum’. The phylogenetic relatedness of PD phytoplasma-infected samples from southern Italy based on *aceF* sequences to each other and to selected strains of the AP-group fruit tree phytoplasmas is depicted in Figure 2. Sample PD3025 of the A12 genotype formed together with reference strain PD-(PD2Luca) a distinct branch and is only distantly related to other samples. Samples of the A8 genotype clustered tightly together and were clearly separated from those of A24, A10 and A12 genotypes.

**Figure 3:**
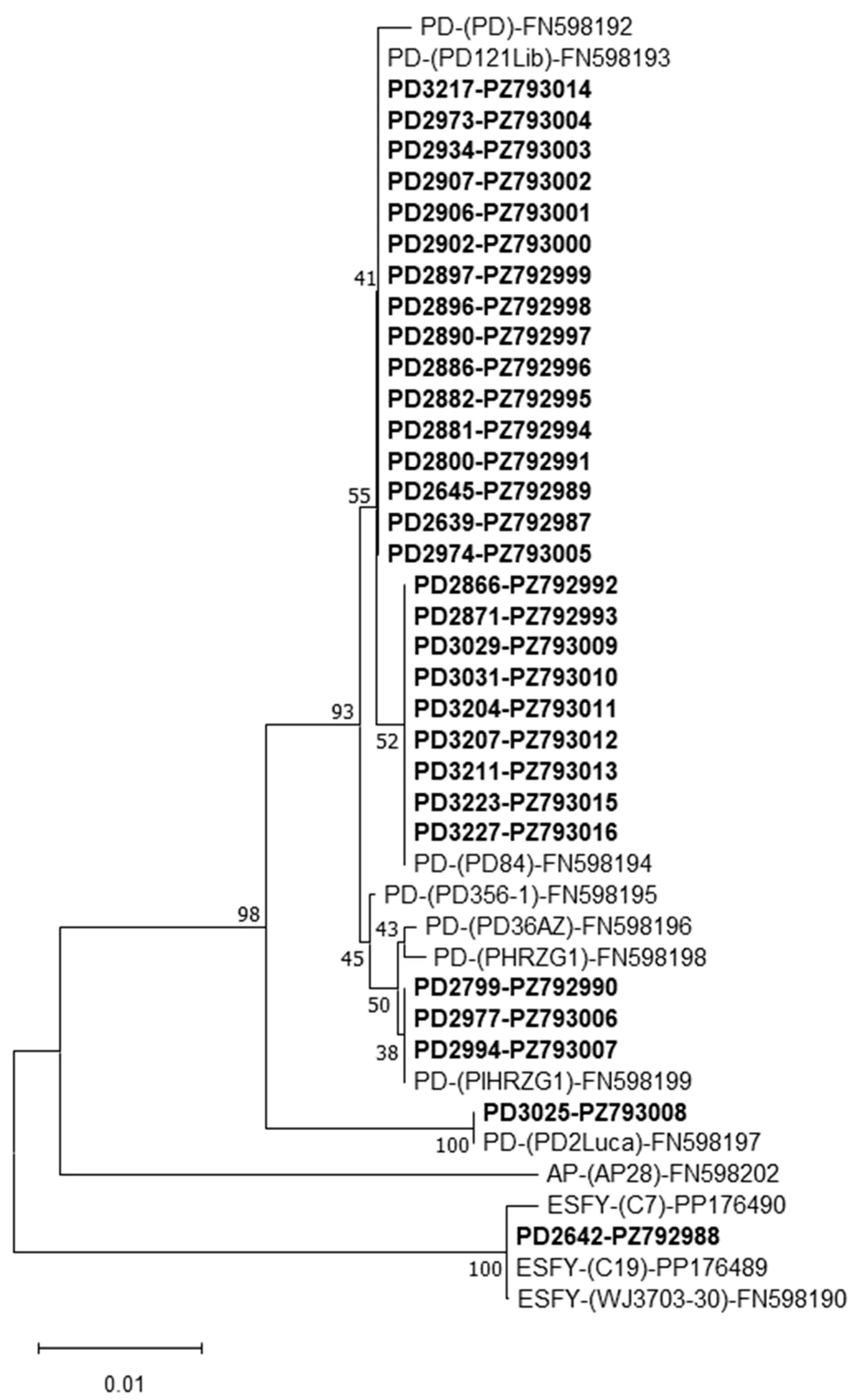
Phylogenetic tree constructed using the neighbor-joining method implemented in MEGA software, version 12 [73] with *pnp* gene sequences from PD phytoplasma-infected samples from southern Italy (in bold type) and related phytoplasma strains. The neighbor-joining tree analysis was conducted using the following parameters: substitution model, (p-distance); gap handling, pairwise deletion; alignment length, 513 bp; trimming criteria, manual trimming using MEGA’s alignment explorer tool. Bar represents a phylogenetic distance of 0.01 nucleotide substitutions per site. GenBank accession number is provided for each phytoplasma-positive sample/strain. Bootstrap values are shown on branches of the phylogenetic tree. See Table S1 for phytoplasma sample/strain abbreviations and further information about the related phytoplasma strains examined.

Comparisons of *pnp* gene sequences revealed the presence of five different genotypes among the 30 PD phytoplasma-infected samples examined (Table 1). These genotypes were P4, P5, P15, P8 and P1 in accordance with previous work by Danet et al. [18]. The P4 genotype was the most abundant in the samples examined and occurred in 15 samples (PD2639, PD2645, PD2800, PD2881, PD2882, PD2886, PD2890, PD2896, PD2897, PD2902, PD2906, PD2907, PD2934, PD2973 and PD2974) from PD-affected pear trees and one sample (PD3217) from PD phytoplasma-infected pear psylla (Table 1). P5 genotype was identified in four samples (PD2866, PD2871, PD3029 and PD3031), which were obtained from PD-affected pear trees, and five samples (PD3204, PD3207, PD3211, PD3223 and PD3227), which were recorded in PD phytoplasma-infected pear psylla. P15 and P8 genotypes were identified in three samples (PD2799, PD2977 and PD2994) and one sample (PD3025), respectively, which were all from PD-affected pear trees. P1 genotype occurred in the sample PD2642, which was taken from PD-affected pear trees. Unlike other *pnp* genotypes identified in the present work, P1 corresponded to the *pnp* gene of ‘*Ca*. Phytoplasma prunorum’ [18]. Genotypes P4, P5, P15 and P8 showed *pnp* sequence similarity, which varied from 97.9 (P5 versus P8) to 99.8% (P4 versus P5). These genotypes shared 94.3–94.7% *pnp* gene sequence similarity with P1 genotype. In phylogenetic analysis of *pnp* gene sequences, PD3025 of the P8 genotype clustered tightly together with the reference phytoplasma strain PD-(PD2Luca) and was clearly separated from samples of P4, P5 and P15 genotypes as well as from PD2442 of P1 genotype. The latter clustered together with reference strains of ‘*Ca*. Phytoplasma prunorum’ (Figure 3).

**Figure 4:**
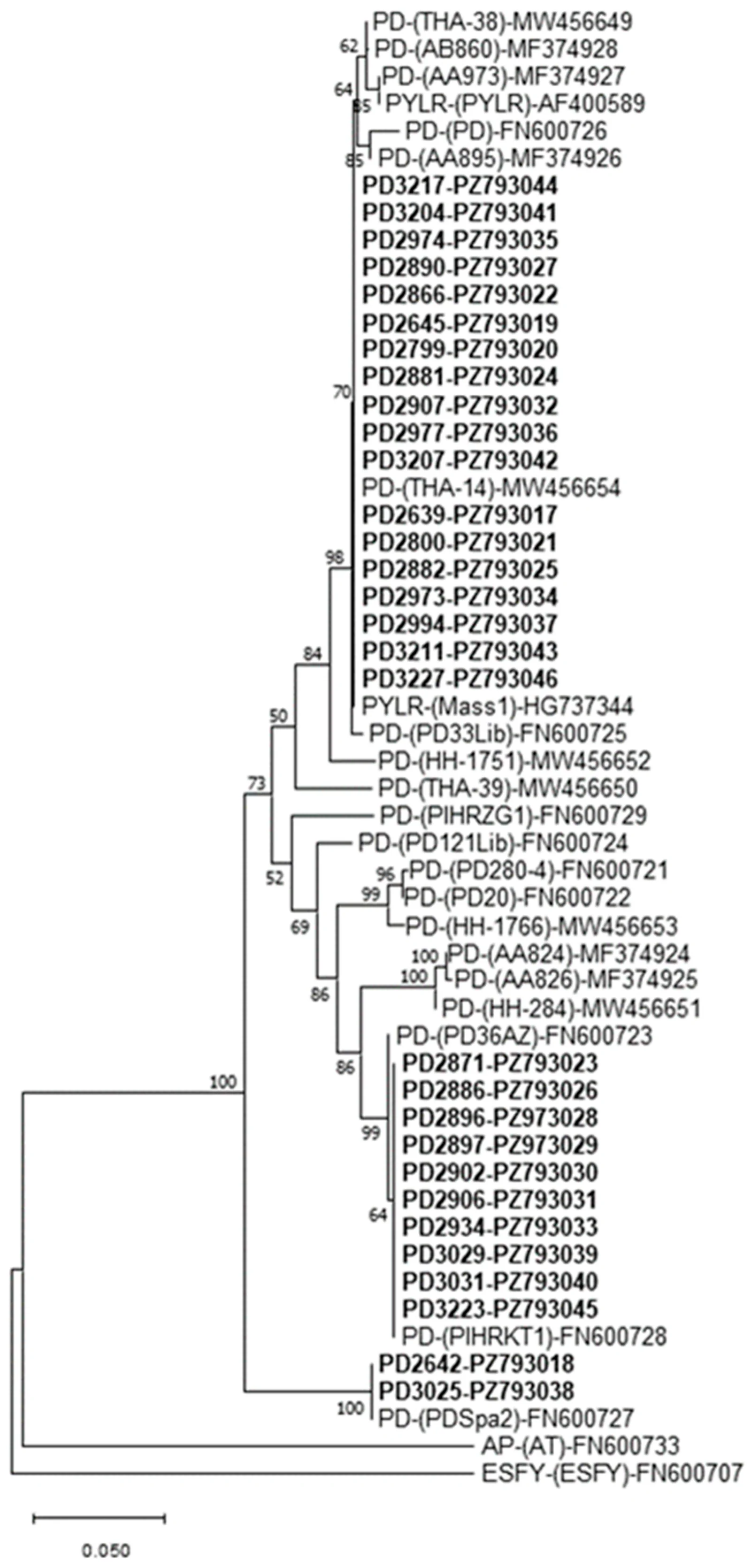
Phylogenetic tree constructed using the neighbor-joining method implemented in MEGA software, version 12 [73] with *imp* gene sequences from PD phytoplasma-infected samples from southern Italy (in bold type) and related phytoplasma strains. The neighbor-joining tree analysis was conducted using the following parameters: substitution model, (p-distance); gap handling, pairwise deletion; alignment length, 464 bp; trimming criteria, manual trimming using MEGA’s alignment explorer tool. Bar represents a phylogenetic distance of 0.05 nucleotide substitutions per site. GenBank accession number is provided for each phytoplasma-positive sample/strain. Bootstrap values are shown on branches of the phylogenetic tree. See Table S1 for phytoplasma sample/strain abbreviations and further information about the related phytoplasma strains examined.

An *imp* genotype indistinguishable from that reported previously in PYLR-affected peach trees in Spain [74], which was later designated as “I40” by Riedle-Bauer et al. [49], was identified in 13 samples (PD2639, PD2645, PD2799, PD2800, PD2866, PD2881, PD2882, PD2890, PD2907, PD2973, PD2974, PD2977 and PD2994) from PD-affected pear trees and five samples (PD3204, PD3207, PD3211, PD3217 and PD3227) from PD phytoplasma-infected pear psylla pools (Table 1). The *imp* genotypes I27 and I20, which were reported previously by Danet et al. [18], occurred in nine samples (PD2871, PD2886, PD2896, PD2897, PD2902, PD2906, PD2934, PD3029 and PD3031) taken from PD-affected pear trees, one sample (PD3223) from PD phytoplasma-infected pear psylla, and in two samples (PD2642 and PD3025) from PD-affected pear trees, respectively. Genotypes I40, I27 and I20 shared *imp* gene sequence similarity, which ranged from 89.4 (I27 versus I20) to 92.3% (I40 versus I27). Phylogenetic analysis of *imp* gene sequences showed that samples PD2642 and PD3025 of the I20 genotype clustered tightly together with reference strain PD-(PDSpa2) and formed a distinct lineage only distantly related to other samples examined. All samples of I40 genotype clustered together along with strains PD-(THA-14), PYLR-(Mass1) and PD-(PD33Lib) and were clearly separated from those of I27 genotype (Figure 4).

**Figure 5:**
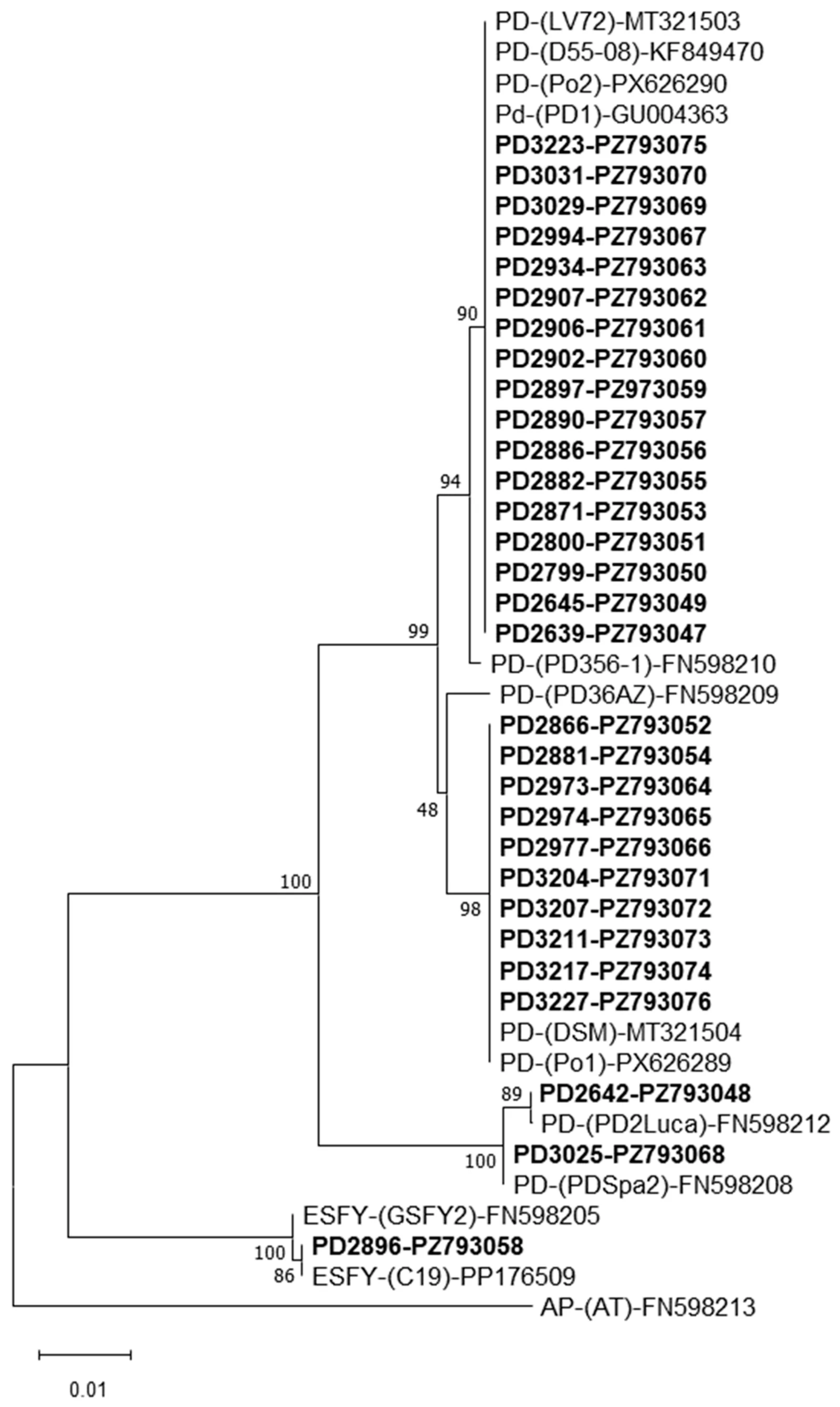
Phylogenetic tree constructed using the neighbor-joining method implemented in MEGA software, version 12 [73] with *SecY* gene sequences from PD phytoplasma-infected samples from southern Italy (in bold type) and related phytoplasma strains. The neighbor-joining tree analysis was conducted using the following parameters: substitution model, (p-distance); gap handling, pairwise deletion; alignment length, 670 bp; trimming criteria, manual trimming using MEGA’s alignment explorer tool. Bar represents a phylogenetic distance of 0.01 nucleotide substitutions per site. GenBank accession number is provided for each phytoplasma-positive sample/strain. Bootstrap values are shown on branches of the phylogenetic tree. See Table S1 for phytoplasma sample/strain abbreviations and further information about the related phytoplasma strains examined.

Five different genotypes were identified at *secY* gene sequence level among the material examined (Table 1). The genotype S13, which was first described in a Slovenian PD phytoplasma strain [46], occurred in 16 samples (PD2639, PD2645, PD2799, PD2800, PD2871, PD2882, PD2886, PD2890, PD2897, PD2902, PD2906, PD2907, PD2934, PD2994, PD3029 and PD3031) from PD-affected pear trees and one sample (PD3223) from PD phytoplasma-infected pear psylla pool (Table 1). PD phytoplasma strains, namely PD1, LV72 and Po2 (Table S1), with *secY* gene sequence identical to that of S13 genotype have previously been detected in Italy [75,76]. However, these strains have not been formally assigned to any *secY* genotype. Five samples from each PD-affected pear tree (PD2866, PD2881, PD2973, PD2974 and PD2977) and PD phytoplasma-infected pear psylla (PD3204, PD3207, PD3211, PD3217 and PD3227) showed a *secY* gene sequence identical to that of PD phytoplasma strains DSM and Po1 (Table S1), which were previously detected in PD-affected pear orchards in Sicily [75]. In continuing and extending the *secY* genotype classification adopted by Danet et al. [18] and Pavšič, et al. [46], it is proposed in the present work that all these entities are assigned to the *secY* genotype S18. Indeed, Pavšič, et al. [46] extended the classification adopted by Danet et al. [18] by proposing new *secY* genotypes, notably S13 through S17. This genotype assignment was not taken into account in a more recent work aimed at classifying strains of ‘*Ca*. Phytoplasma mali’ [77,78]. *SecY* genotypes S8 and S4 [18] were identified in the samples PD2642 and PD3025, respectively, which were both taken from PD-affected pear trees. The *secY* genotype S1 [18] was identified in the sample PD2896 from PD-affected pear trees. Unlike other *secY* genotypes identified in the present work, S1 corresponded to the *secY* gene of ‘*Ca*. Phytoplasma prunorum’ [18]. Genotypes S13, S18, S8 and S4 showed *secY* gene sequence similarity, which varied from 96.0 (S13 versus S8) to 99.8% (S13 versus S4). These genotypes had 92.7–92.9% *secY* gene sequence similarity with S1 genotype. In phylogenetic analysis of *secY* gene sequences, PD phytoplasma-infected samples from southern Italy, which were assigned to S13 genotype, clustered tightly together with the PD phytoplasma strains from Slovenia (D55-08) and Italy (PD1, LV72 and Po2), whereas those assigned to S18 genotype clustered together with PD phytoplasma strains DSM and Po1, which were previously detected in PD-affected pear orchards in Sicily (Figure 5). The remaining samples PD2642, PD3025 and PD2896 of *secY* genotypes S8, S4 and S1, respectively, recorded in the present work each formed distinct branches in the phylogenetic tree (Figure 5).

### 2.5. Multilocus Profile Identification and Phylogenetic Analysis of the Concatenated Sequences

**Figure 6:**
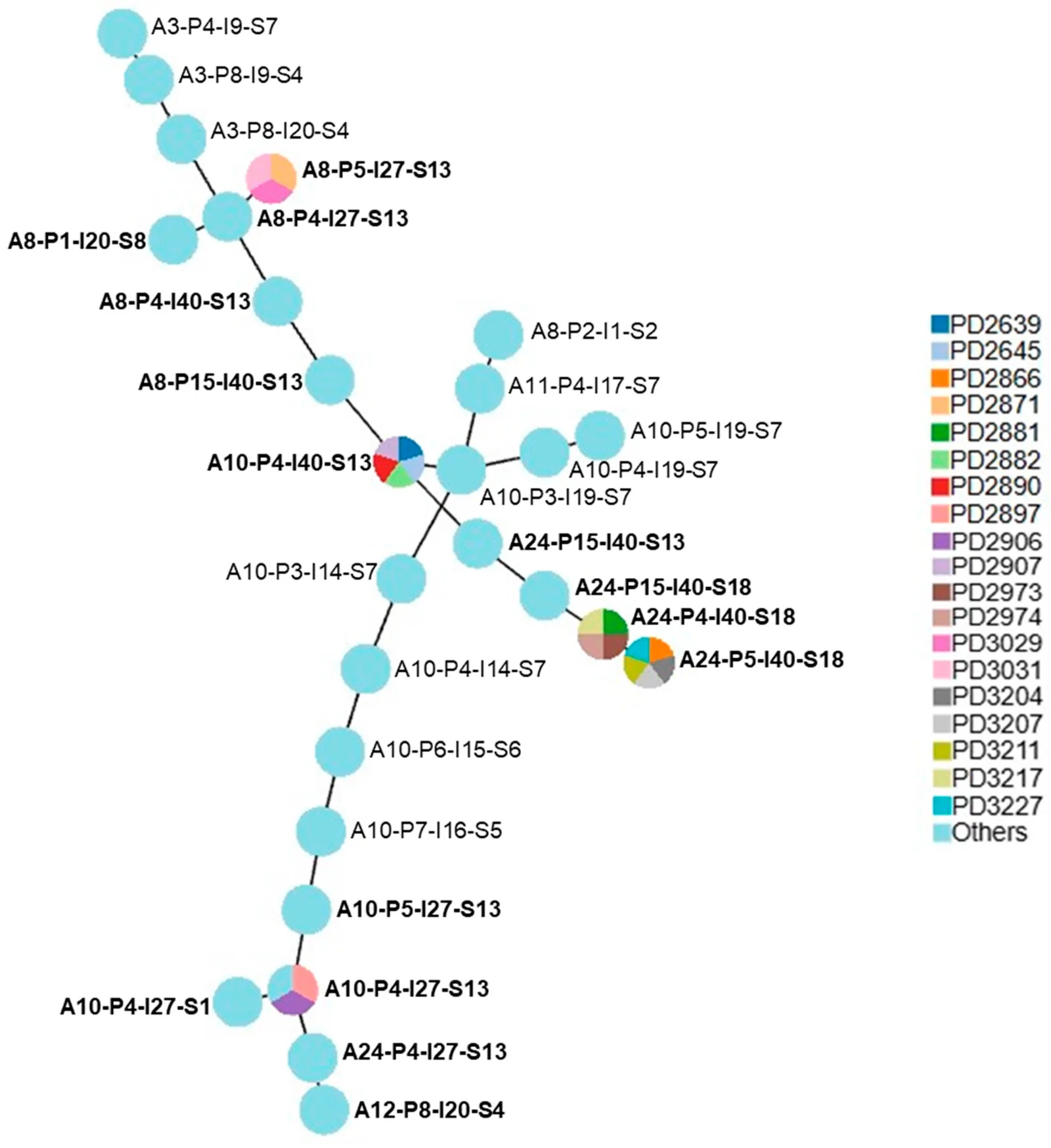
Similarity network of multilocus profiles obtained from the 30 selected PD phytoplasma-infected samples examined in southern Italy (in bold type) and previously published profiles [18]. Profiles are defined by the allelic designations at the *aceF*, *pnp*, *imp* and *secY* loci. The network was constructed using the goeBURST (global optimal eBURST) algorithm, a refinement of the eBURST algorithm [79], implemented in the PHYLOViZ 2.0 software [80] (http://online.phyloviz.net, accessed on 20 July 2026). Clonal complexes (CCs) were defined at the single-locus-variant (SLV). Node size represents the number of samples assigned to each profile in the dataset analyzed. See Table 1 for profile designations.

A total of 15 different multilocus profiles were identified among the selected 30 PD phytoplasma-infected samples examined in southern Italy (Table 1). Of these, profiles A10-P4-I40-S13 and A24-P5-I40-S18 proved to be the most common, each occurring in five samples. Multilocus profiles A24-P5-I40-S18 and A24-P4-I40-S18 occurred in both samples from PD-affected pear trees and PD phytoplasma-infected pear psylla. Profile A10-P5-I27-S13 was identified only in the sample PD3223 from PD phytoplasma-infected pear psylla, whereas the remaining profiles were identified only in samples from PD-affected pear trees. All ‘*Ca*. Phytoplasma pyri’ profiles identified in the present study differed from those previously published [18]. The relationships among the 15 multilocus profiles identified in this study and the published profiles included for comparison are shown in Figure 6.

**Figure 7:**
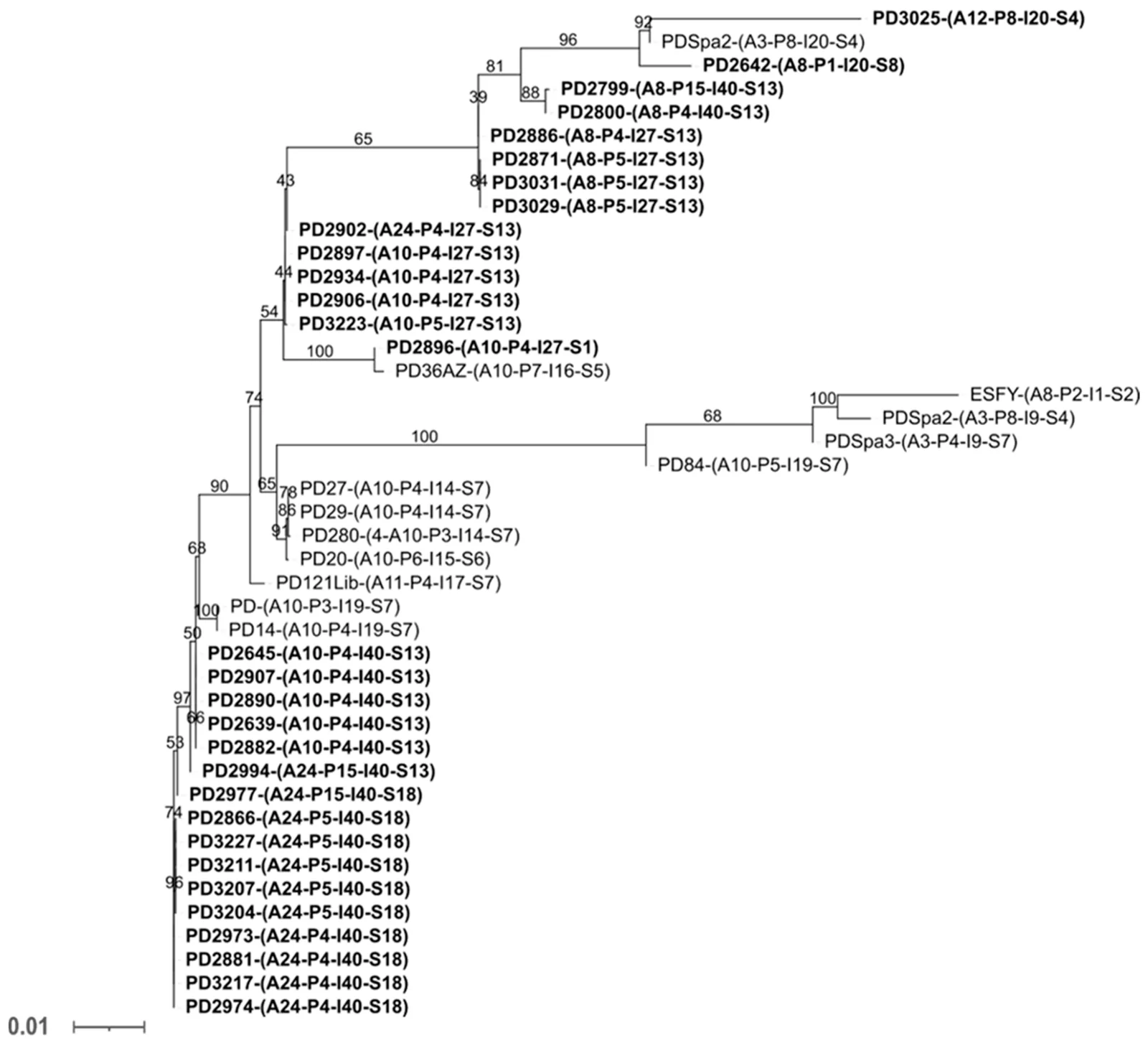
Phylogenetic tree constructed using concatenated *aceF*, *pnp*, *imp*, and *secY* gene sequences from the 30 selected PD phytoplasma-infected samples from southern Italy (shown in bold) and related phytoplasma strains for which multilocus profiles based on the four genes were available. Maximum likelihood (ML) analysis was performed using IQ-TREE version 3.1.4 [81], with gene-specific substitution models selected by ModelFinder according to the Bayesian Information Criterion (BIC): HKY + F + I for *aceF*, TPM2u + F + I for *pnp*, TPM3u + F + G4 for *imp*, and HKY + F + I for *secY*. Branch support was assessed using 1000 ultrafast bootstrap replicates. The scale bar represents 0.01 nucleotide substitutions per site. Bootstrap values are shown at the corresponding branches. See Table 1 for multilocus profile designations.

Phylogenetic relationships inferred from the concatenated and partitioned *aceF*, *pnp*, *imp* and *secY* gene sequences are depicted in Figure 7. Most of the profiles of the samples examined clustered together along with those of reference strains PD-(A10-P3-I19-S7) and PD14-(A10-P4-I19-S7). Profiles of samples PD3025-(A12-P8-I20-S4) and PD2642-(A8-P1-I20-S8) showed branching patterns different from those of other samples.

Mitochondrial 16S rDNA sequences of the amplicons obtained with primer pair 16SR21/16S22 from two pools of pear psylla examined proved to be identical to each other and to that of *Cacopsylla pyri* available in the GenBank database (GenBank accession no. MK039582).

## 3. Discussion

Prior to this work, PD disease has been reported in the neighboring regions Campania, Basilicata, Calabria and Apulia of southern Italy [50,66,67,68,69,70,71]. However, several aspects of this disease such as extent of occurrence and impact, relationships with insect vector, and genetic variability of the causal agent were not investigated, or there is sparse information on these aspects.

In the current study, visual symptoms assessment and PCR assays using rDNA-directed primers, followed by RFLP analysis, were employed to update and extend knowledge on the occurrence of PD disease in southern Italy in several pear-growing areas, including those which had previously not been examined. For detection and identification of PD phytoplasma as well as other fruit tree phytoplasmas of the AP group by PCR assays, universal, group- and pathogen-specific primers have been designed, directed to both ribosomal and non-ribosomal DNA sequences [1,46,82,83,84,85,86]. Primers amplifying rDNA sequences are being used extensively because of their satisfactory sensitivity. However, there are reports on difficulties to differentially detect the various fruit tree phytoplasmas of the AP group, notably the PD agent, using pathogen-specific primers Ribosomal primers designed for specific detection of the PD phytoplasma cross-amplified DNA from other AP-group fruit tree phytoplasmas, especially AP and ESFY agents, due to the close relationship of these taxa at the rDNA sequence level [1,42,81,82,83]. In addition, pathogen-specific primers failed to detect all strains of the PD agent [1,83,85]. Thus, it is advisable to use group-specific primers that amplify all AP-group fruit tree phytoplasmas and all strains of each of these pathogens. Reliable identification of the PD phytoplasma can be achieved through RFLP analysis of PCR-amplified 16S rDNA using suitable restriction enzymes [42,83,85]. With this method, the PD agent is clearly distinguished from AP and ESFY phytoplasmas. By employing PCR assays with AP group-specific primers and a primer pair that preferentially amplifies a 16S rDNA fragment from the PD phytoplasma, followed by RFLP analysis, we detected the PD phytoplasma in all symptomatic pear trees examined and in 46 of the 63 pools of *Cacopsylla pyri* insects captured in the surveyed pear orchards. However, 25 of the 181 phytoplasma-positive pear trees (13.8%) and five of the 46 phytoplasma-positive insect pools (10.9%) yielded detectable amplification products only after nested PCR. This may be due to several factors including the extremely low titer of the infecting phytoplasma and its uneven distribution in the examined tissues [87,88].

Disease incidence of PD was different in the pear orchards examined. Serious PD incidence was encountered in the orchards located near Battipaglia and Pontecagnano Faiano, where a high percentage of pear trees (65%) appeared to be affected and showed severe symptoms. In all affected pear orchards examined, the most characteristic symptom observed was reddening of the foliage. This symptom appeared mostly as early as mid-July to reach its peak in late summer and fall. Diseased pear trees showing pronounced foliar reddening symptoms yielded strong PCR signals and/or tested phytoplasma positive by direct PCR. This is clear evidence that pear trees that were intensively colonized in the aerial parts by the invading pathogen usually developed characteristic symptoms such as foliar reddening in accordance with previous findings [1]. Reddening of the foliage in late summer and fall may also occur on healthy pear trees grown on quince rootstocks, indicating incompatibility problems. However, reddening of healthy trees on quince appears several weeks later in the season than reddening due to PD phytoplasma infections [1,89]. Reddening of healthy trees on quince was not observed in the present work. Although the quick decline syndrome is prevalent on trees on oriental rootstocks *P. pyrifolia* and *P. ussuriensis* and to a lesser extent on *P. communis* rootstocks, some slow decline-affected pear trees recorded in the present study suddenly wilted and died in summer in the presence of abnormally hot and dry weather conditions. It is well known that these conditions are predisposing factors for quick decline [1] and that they are currently common in summer, especially in southern Italy. Previous studies showed that pear trees grown on quince rootstocks in Germany were little affected by the PD phytoplasma, owing to the poor host properties of quince. In this rootstock, the PD phytoplasma occurred in low titer, which had a negative effect on the seasonal recolonization of the aerial parts that usually takes place in spring. Thus, it is usually rated as fairly resistant (for references, see [1]). In contrast, pear trees on quince rootstocks were found to be severely affected by PD phytoplasma in various pear-growing areas of central and northern Italy [90,91]. These differences were attributed to the fact that psyllid infestation was heavier in central and northern Italy than in Germany. Thus, the top of trees in central and northern Italy is repeatedly reinfected and more heavily than when recolonization starts from roots [1,92,93]. In the present study, severe PD symptoms were observed in pear trees grafted onto BA29 quince rootstock. However, the survey was not designed to disentangle the effects of tree age, cultivar, orchard conditions, management practices and vector abundance. The contribution of these factors to the observed symptom severity therefore remains unresolved.

*Cacopsylla pyri,* previously documented in Campania and elsewhere in southern Italy [94,95,96], was the only *Cacopsylla* species collected in the three monitored pear orchards in Campania. Phytoplasma DNA was detected in 46 of the 63 insect pools examined (73.0%). This percentage represents the proportion of positive pools and cannot be interpreted as the proportion of infected individual insects, whereas the estimated individual infection probability was 12.0% (95% exact confidence interval: 8.8–16.5%). The transmission competence of *C. pyri* for *‘Ca.* Phytoplasma pyri’ has already been established experimentally, including in Italy [62,97].

Data obtained from multilocus sequence typing (MLST) analysis of *aceF*, *pnp*, *imp* and *secY* gene sequences revealed a considerable genetic variability among 30 selected PD phytoplasma-infected samples examined in southern Italy. The highest number of genotypes was observed for *pnp* and *secY* genes (five genotypes for each), whereas the highest sequence dissimilarity value between the samples under study (5.3%) occurred in the *aceF* gene. For each gene examined, the most frequently identified genotypes were A24, P4, I40 and S13, respectively. All were identified in samples from both PD-affected pear trees and PD phytoplasma-infected pear psylla. The less frequently identified genotypes were A12, P8, P1, I20, S8, S4 and S1. These genotypes, with the exception of P8, occurred only in one sample from pear trees. Genotype A8, which was identified in the samples PD2642, PD2799, PD2800, PD2871, PD2886, PD3029 and PD3031; genotype P1, which occurred in the sample PD2642; and genotype S1, which was identified in the sample PD2896, corresponded to *aceF*, *pnp*, and *secY* gene sequences of ‘*Ca*. Phytoplasma prunorum’, respectively. The detection of alleles reported previously in ‘*Ca*. Phytoplasma prunorum’ in samples identified as ‘*Ca*. Phytoplasma pyri’ is consistent with previous observations interpreted as supporting possible interspecies recombination [18,48,51,52,53]. However, the multilocus profiles obtained in the present study do not conclusively demonstrate recombination. Mixed infections, preferential amplification of different templates at different loci, and PCR-mediated recombination cannot be excluded using the methods employed. In particular, the 16S rRNA gene RFLP profiles were consistent with ‘*Ca.* Phytoplasma pyri’ but did not exclude a minor component of another phytoplasma species. The observed multilocus profiles should therefore be regarded as compatible with the hypothesis of possible interspecies recombination, pending further investigation to establish whether the alleles occur within the same phytoplasma genome.

Fifteen distinct multilocus profiles were identified in the 30 selected PD phytoplasma-infected samples recorded in southern Italy. These profiles differed from those reported previously by Danet et al. [18], and their similarities were visualized in a network. The network provides a descriptive representation of the profiles included in the analysis but does not establish their ancestry or evolutionary origin. Interpretation is also limited by the selection of samples and the availability of published profiles. Furthermore, because insect samples were analyzed as pools and amplicons were sequenced directly, the allelic combinations obtained from these samples cannot be unequivocally assigned to individual phytoplasma genomes. Additional sampling and analyses resolving individual infections would be needed to investigate the population structure and evolutionary relationships of the detected phytoplasmas.

## 4. Materials and Methods

### 4.1. Plant Samples, Symptom Evaluation and Phytoplasma Reference Strains

Pear trees showing PD symptoms were sampled from 2023 through 2025, in particular during late summer and autumn when foliar symptoms were more pronounced, in different commercial orchards located near Battipaglia (two orchards, in the following referred to as Bat 1 and Bat 2, 35 and 30 trees, respectively), Pontecagnano Faiano (orchard Pont, 22 trees), Caserta (orchard Cas, 19 trees) (all Campania region), near Paterno (orchard Pat, 34 trees) and Grumento Nova (orchard Gru, 23 trees) in the Agri valley of the Basilicata region, and near Sibari (orchard Sib, 18 trees) in the Sibari plain of the Calabria region. The pear orchards were conventionally managed and varied in size from 1 to 5 ha. The trees were less than 12 years old, grown on quince rootstocks BA29, and grafted with cvs. Etrusca, Coscia and Williams. Most of these trees, notably all trees that tested phytoplasma positive only through nested PCR assays, were sampled twice for re-examination throughout the mentioned sampling period. Also, trees were kept under observation throughout the mentioned 3-year period for evaluation of symptom development. Disease incidence was calculated as the percentage of pear trees showing PD symptoms relative to the total number of pear trees present in each orchard, which were assessed using the formula Disease incidence = (No. of symptomatic trees/total no. of trees assessed) x 100. A number of non-symptomatic pear trees randomly selected from each pear orchard surveyed were also included in this study for examination. These trees were sampled in the same period as symptomatic trees. In particular, 7, 6, 5, 4, 7, 5, and 4 non-symptomatic trees were sampled in the mentioned orchards, respectively. Healthy pear seedlings kept in an insect-proof greenhouse were used as healthy plant controls. In addition, DNA samples from the phytoplasma reference strains AT of the AP phytoplasma, PD1 of the PD phytoplasma, and GSFY1 of the ESFY agent, which were previously transmitted to periwinkle and maintained in this host by periodic grafting, were used for comparison [59,71].

### 4.2. Psylla Collection

Pear psyllids were collected through yellow sticky traps (24.5 × 13.5 cm, Biogard, Bergamo, Italy), hung in the tree canopy at about 1.5 m from the ground, in the three above-mentioned pear orchards located in the Campania region near Battipaglia (orchards Bat 1 and Bat 2) and Pontecagnano Faiano (orchard Pont) from 2024 to 2025 (Figure S1). Seven traps in each orchard Bat 1 and orchard Pont and 12 traps in orchard Bat 2 were collected and replaced every two weeks or when dirt or debris interfered with their effectiveness from mid-May to mid-October. Pear psyllids were removed from traps by separating them from other fauna, identified using different identification keys [98,99], counted and stored under 95% ethanol until tested for phytoplasma infections. Species identification was based on diagnostic morphological characters, including the morphology of both male and female genitalia, using the identification keys cited above.

### 4.3. DNA Extraction and PCR Analysis

Total DNA was extracted from approximately 1.0 g of fresh tissue taken from diseased and healthy pear trees that was processed following the phytoplasma-enrichment procedure previously described by Ahrens and Seemüller [100]. Fresh tissue consisted of leaf petioles, midribs and phloem scrapings. The latter were prepared from current season’s shoots and occasionally from several-year-old stem portions or roots according to Ahrens and Seemüller [101]. Total DNA from pear psylla was extracted from groups of 10 individuals using a CTAB-based extraction procedure as described previously [102]. Since phytoplasma detection by the highly sensitive nested PCR assays bears the risk that contaminations are amplified, an extraction blank control containing all the extraction reagents was processed identically to real biological samples, running it through every single step of the extraction procedure either from plant tissue or pear psylla.

For PCR analysis, both ribosomal and non-ribosomal DNA (rDNA) primers were used. All primers used and their sequences are shown in Table S2. Universal phytoplasma primer pair P1/P7 [103] amplifies a 1850 bp fragment that extends from the 5′ end of the 16S rRNA gene to the 5′ region of the 23S rDNA. Primer pair fO1/rO1 specifically amplifies a 16S rDNA fragment of about 1070 bp in length from all fruit tree phytoplasmas of the AP group [83]. Primer fPD combined with primer rO1 preferentially amplifies a 900 bp 16S rDNA fragment from the PD phytoplasma [83,85]. In nested PCR assays, amplicons obtained with primer pair P1/P7 were re-amplified with either primer pair fO1/rO1 or with fPD/rO1. For amplification of non-rDNA sequences, the following primer pairs were used: AceFf1/AceFr1, followed by AceFf2/AceFr2, which amplifies a DNA fragment of about 790 bp in length of the *aceF* gene from fruit tree phytoplasmas of the AP group [18]; Pnpf1/Pnpr1, followed by Pnpf2/Pnpr2, which amplifies a 550 bp DNA fragment of the *pnp* gene from fruit tree phytoplasmas of the AP group [18]; IMPF2bis/IMPR1bis, followed by IMPF3pyr/IMPR4pyrA, which amplifies a 450 bp DNA fragment of the *imp* gene from the PD phytoplasma [18]; and SecYMalF1/SecYMalR1, followed by SecYMalF2/SecYMalR2, which amplifies a DNA fragment of about 660 bp in length of the *secY* gene from AP group-fruit tree phytoplasmas [18]. Mitochondrial 16S rDNA sequences of the pear psylla were amplified using the primer pair 16SR21/16S22. These primers amplify a fragment of approximately 450 bp in length [104].

PCR amplifications were carried out in a 50 µL reaction mixture containing 125 µM of the four dNTPs, 0.5 µM of each primer, 1U of Dream Taq DNA polymerase, 1x polymerase buffer (both Thermo Fisher Scientific, Waltham, MA, USA), 5 µL of template DNA (100–200 ng), and water, using the instrument Applied Biosystems VeritiPro 96-Well Thermal Cycler (Thermo Fisher Scientific, Waltham, MA, USA). The reaction mixture was subjected to 35 cycles at the following conditions: 30 s denaturation at 95 °C, 60 s annealing at 55 °C (50 °C with non-rDNA primers), and 90 s extension (60 s with the primer pairs fO1/rO1 and fPD/rO1 and with non-rDNA primers) at 72 °C. The final step was 10 min at 72 °C. Nested PCR was carried out using 1 μL of PCR products obtained in the initial amplification as template in the reaction mixture described above. Reaction mixture devoid of DNA template was included in all PCR assays as a negative control. Also, the presence of PCR inhibitory factors in DNA extracts from pear trees and psylla that proved to be phytoplasma negative was tested by adding small amounts of DNA preparation (amplification inhibition control) from the phytoplasma reference strains used for comparison. Five µL of PCR products was analyzed by gel electrophoresis in a 1.5% horizontal agarose gel in TAE buffer (40 mM Tris-acetate, 1 mM EDTA, pH 8.0) in the presence of 0.5 µg/mL SYBR Safe DNA gel stain (Invitrogen, Waltham, MA, USA). DNA bands were visualized through the ImageQuant LAS 4000 system, version 1.2 (GE Healthcare Bio-Sciences AB, Uppsala, Sweden).

### 4.4. RFLP Analysis of PCR Products

Ten microliters of products obtained in direct or nested PCR with primer pair fO1/rO1 was digested separately with SspI and RsaI restriction endonucleases following the manufacturer’s instructions (Thermo Fisher Scientific). Five microliters of the digests was used to resolve the restriction fragments in a 3% horizontal agarose gel in TAE and/or on vertical 5% polyacrylamide gels in TBE buffer (45 mM Tris-borate, 1 mM EDTA, pH 8). After electrophoresis, the DNA was visualized with SYBR Safe DNA gel stain as described above. Molecular weights were determined using the 1 Kb Plus DNA ladder (Thermo Fisher Scientific).

### 4.5. Sequencing of DNA and Data Analysis

PCR products obtained with primer pairs AceFf2/AceFr2, Pnpf2/Pnpr2, IMPF3pyr/IMPR4pyrA and SecYMalF2/SecYMalR2 from 24 PD-infected pear tree samples and 6 phytoplasma-positive *C. pyri* pools were purified using the PureLink PCR Purification Kit (Invitrogen, Waltham, MA, USA) and sequenced directly. Allelic designations were assigned to the sequences obtained by direct amplicon sequencing, and their combinations were recorded as sample-level multilocus profiles. Amplicons for sequencing were obtained from positive samples that were selected among those with the highest quality and intensive PCR products from each surveyed orchard (Table 1). Sequencing of both strands of each PCR-amplified fragment was carried out by BMR Genomics, University of Padua, Padua, Italy, using the same primers employed in PCR analysis. The obtained sequences were assembled and edited using either DNASTAR’s LaserGene software (DNASTAR), version 7.1 or the sequence analysis software BioEdit, version 7.2.5 [105]. They were then used in a BLAST, version 2.17.0 search analysis carried out online at https://blast.ncbi.nlm.nih.gov/Blast.cgi, (accessed on 20 July 2026) [106]. Multiple sequence alignments were performed using CLUSTAL W of the LaserGene software (DNASTAR) or of the BioEdit software (BioEdit). Sequence similarities were calculated through the MEGALIGN program of the LaserGene software (DNASTAR). GenBank accession nos. of deposited sequences are as follows: PZ792957 to PZ792986 for *aceF*, PZ792987 to PZ793016 for *pnp*, PZ793017 to PZ793046 for *imp*, PZ793047 to PZ793076 for *secY*, and QB058825 to QB058826 for mitochondrial 16S rRNA genes. Other sequences that were retrieved from the GenBank database and used in this study for comparisons are given in Table S1 and Figure 2, Figure 3, Figure 4 and Figure 5.

Phylogenetic and molecular evolutionary analyses were carried out with the genetic analysis software MEGA, version 12 using the neighbor-joining parameters and the bootstrap test [73]. The data were resampled 1000 times, and the bootstrap percentage values are given at the nodes of the tree. Neighbor-joining and maximum likelihood methods yielded similar results in tree construction. The maximum-likelihood tree analysis in MEGA software was conducted using the following parameters: substitution model, Tamura-Nei; rates among sites, uniform rates; initial tree for ML analysis, make initial tree automatically via NJ/MP; ML Heuristic Method, Nearest-Neighbor-Interchange (NNI), test of phylogeny, bootstrap method; bootstrap replicates, 1000. Multilocus sequence network was constructed by analyzing the concatenated *aceF*, *pnp*, *imp* and *secY* gene sequences of each sample using the goeBURST (global optimal eBURST) algorithm, a refinement of the eBURST algorithm [79], implemented in the PHYLOViZ 2.0 software [80], and was based on already published multilocus profiles and multilocus profiles identified in this work. Clonal complexes (CCs) were defined at the single-locus-variant (SLV). The *aceF*, *pnp*, *imp*, and *secY* gene sequences, aligned individually and concatenated using Concatenator software v. 0.3.1 [107], were subjected to maximum likelihood (ML) phylogenetic analysis using IQ-TREE version 3.1.4 [81]. The concatenated alignment comprised four gene-specific partitions, one for each gene. The best-fitting nucleotide substitution model for each partition was selected using ModelFinder according to the Bayesian Information Criterion (BIC). The selected models were HKY + F + I for *aceF*, TPM2u + F + I for *pnp*, TPM3u + F + G4 for *imp*, and HKY + F + I for *secY*. An edge-linked proportional partition model was applied, allowing separate substitution models and evolutionary rates across partitions while linking branch lengths proportionally among partitions. Branch support was assessed using 1000 ultrafast bootstrap replicates. The resulting phylogenetic tree was visualized using iTOL v. 7 [108]. The individual gene alignments, concatenated partitioned alignment, and final tree in Newick format are provided as Supplementary Data.

### 4.6. Statistical Estimation of Infection Rates of Pear Psylla

The infection rate (p) of the captured pear psylla group tested by PCR assays was estimated by the maximum likelihood estimator of p using the formula p = 1 − H^1/k^, where H is the proportion of negative pools out of the total pools tested and k is the pool size (the number of insects grouped together in a single tube) [109]. The 95% confidence interval was calculated by first obtaining the exact binomial Clopper–Pearson confidence limits for the proportion of positive pools (46/63) and then transforming both limits to the individual level using (p = 1 − (1 − q)^(1/k)^), where (q) is the proportion of positive pools. This yielded a 95% confidence interval of 8.8–16.5% [110].

## 5. Conclusions

This study documents the occurrence of PD-associated phytoplasma in symptomatic pear trees sampled in selected orchards in Campania, Basilicata and Calabria. PCR assays, followed by RFLP analysis, detected ‘*Ca.* Phytoplasma pyri’ in all 181 symptomatic trees examined. Severe symptoms were observed in trees grafted onto BA29 quince rootstock, although the relative contributions of cultivar, tree age, orchard conditions, management and vector abundance were not assessed.

*Cacopsylla pyri* was the only species collected in the three monitored orchards in Campania, and 46 of the 63 insect pools examined tested phytoplasma positive. These findings support the likely involvement of this established vector in local pathogen spread, without directly assessing the infectivity or transmission efficiency of the sampled insects.

Multilocus analysis of *aceF*, *pnp*, *imp* and *secY* revealed 15 distinct profiles among the 30 selected PD phytoplasma-infected samples, comprising 24 plant samples and six insect pools. Some samples yielded alleles reported previously in ‘*Ca.* Phytoplasma prunorum’, resulting in profiles compatible with the hypothesis of possible interspecies recombination. However, mixed infection, preferential amplification and PCR-mediated recombination are not excluded, and the observed allelic combinations cannot be unequivocally assigned to individual phytoplasma genomes. The similarity network describes relationships among the profiles examined without establishing their ancestry. These results extend knowledge of PD occurrence and the multilocus diversity detected in the sampled orchards.

## Figures and Tables

**Table 1:** Genotyping data and other details of pear decline (PD) phytoplasma-positive samples recorded in southern Italy and related phytoplasma strains.

Sample/Strain	Genotype *				Multilocus Profile †	Original Host	Orchard	Reference
	*aceF*	*pnp*	*imp*	*secY*				
PD2639	A10	P4	I40	S13	A10-P4-I40-S13	*Pyrus communis*	Bat 1	[This paper]
PD2642	A8	P1	I20	S8	A8-P1-I20-S8	*Pyrus communis*	Bat 1	[This paper]
PD2645	A10	P4	I40	S13	A10-P4-I40-S13	*Pyrus communis*	Bat 1	[This paper]
PD2799	A8	P15	I40	S13	A8-P15-I40-S13	*Pyrus communis*	Bat 1	[This paper]
PD2800	A8	P4	I40	S13	A8-P4-I40-S13	*Pyrus communis*	Bat 2	[This paper]
PD2866	A24	P5	I40	S18 **	A24-P5-I40-S18	*Pyrus communis*	Bat 2	[This paper]
PD2871	A8	P5	I27	S13	A8-P5-I27-S13	*Pyrus communis*	Bat 2	[This paper]
PD2881	A24	P4	I40	S18 **	A24-P4-I40-S18	*Pyrus communis*	Bat 2	[This paper]
PD2882	A10	P4	I40	S13	A10-P4-I40-S13	*Pyrus communis*	Pont	[This paper]
PD2886	A8	P4	I27	S13	A8-P4-I27-S13	*Pyrus communis*	Pont	[This paper]
PD2890	A10	P4	I40	S13	A10-P4-I40-S13	*Pyrus communis*	Pont	[This paper]
PD2896	A10	P4	I27	S1	A10-P4-I27-S1	*Pyrus communis*	Cas	[This paper]
PD2897	A10	P4	I27	S13	A10-P4-I27-S13	*Pyrus communis*	Cas	[This paper]
PD2902	A24	P4	I27	S13	A24-P4-I27-S13	*Pyrus communis*	Cas	[This paper]
PD2906	A10	P4	I27	S13	A10-P4-I27-S13	*Pyrus communis*	Pat	[This paper]
PD2907	A10	P4	I40	S13	A10-P4-I40-S13	*Pyrus communis*	Pat	[This paper]
PD2934	A10	P4	I27	S13	A10-P4-I27-S13	*Pyrus communis*	Pat	[This paper]
PD2973	A24	P4	I40	S18 **	A24-P4-I40-S18	*Pyrus communis*	Pat	[This paper]
PD2974	A24	P4	I40	S18 **	A24-P4-I40-S18	*Pyrus communis*	Gru	[This paper]
PD2977	A24	P15	I40	S18 **	A24-P15-I40-S18	*Pyrus communis*	Gru	[This paper]
PD2994	A24	P15	I40	S13	A24-P15-I40-S13	*Pyrus communis*	Gru	[This paper]
PD3025	A12	P8	I20	S4	A12-P8-I20-S4	*Pyrus communis*	Sib	[This paper]
PD3029	A8	P5	I27	S13	A8-P5-I27-S13	*Pyrus communis*	Sib	[This paper]
PD3031	A8	P5	I27	S13	A8-P5-I27-S13	*Pyrus communis*	Sib	[This paper]
PD3204	A24	P5	I40	S18 **	A24-P5-I40-S18	*Cacopsylla pyri*	Bat 1	[This paper]
PD3207	A24	P5	I40	S18 **	A24-P5-I40-S18	*Cacopsylla pyri*	Bat 1	[This paper]
PD3211	A24	P5	I40	S18 **	A24-P5-I40-S18	*Cacopsylla pyri*	Bat 2	[This paper]
PD3217	A24	P4	I40	S18 **	A24-P4-I40-S18	*Cacopsylla pyri*	Bat 2	[This paper]
PD3223	A10	P5	I27	S13	A10-P5-I27-S13	*Cacopsylla pyri*	Pont	[This paper]
PD3227	A24	P5	I40	S18 **	A24-P5-I40-S18	*Cacopsylla pyri*	Pont	[This paper]
PD	A10	P3	I19	S7	A10-P3-I19-S7	*Pyrus communis*		[18]
ESFY	A8	P2	I1	S2	A8-P2-I1-S2	*Prunus armeniaca*		[18]
PD36AZ	A10	P7	I16	S5	A10-P7-I16-S5	*Pyrus communis*		[18]
PD14	A10	P4	I19	S7	A10-P4-I19-S7	*Pyrus communis*		[18]
PDSpa2 ***	A3	P8	I20	S4	A3-P8-I20-S4	*Pyrus communis*		[18]
PDSpa2 ***	A3	P8	I9	S4	A3-P8-I9-S4	*Pyrus communis*		[18]
PDSpa3	A3	P4	I9	S7	A3-P4-I9-S7	*Pyrus communis*		[18]
PD280-4	A10	P3	I14	S7	A10-P3-I14-S7	*Pyrus communis*		[18]
PD20	A10	P6	I15	S6	A10-P6-I15-S6	*Pyrus communis*		[18]
PD27	A10	P4	I14	S7	A10-P4-I14-S7	*Pyrus communis*		[18]
PD29	A10	P4	I14	S7	A10-P4-I14-S7	*Pyrus communis*		[18]
PD84	A10	P5	I19	S7	A10-P5-I19-S7	*Pyrus communis*		[18]
PD121Lib	A11	P4	I17	S7	A11-P4-I17-S7	*Pyrus communis*		[18]

* Genotype designation according to Danet et al. [18]; Pavšič et al. [46]; Riedle-Bauer et al. [49]; Kiss et al. [72]. ** Genotype designation newly proposed in this study. *** Strain for which two different *imp* gene sequences were detected, one (I20) corresponding to ‘*Ca*. Phytoplasma pyri and one (I9) corresponding to ‘*Ca*. Phytoplasma prunorum [18]. † Multilocus profiles represent combinations of alleles detected in each PD phytoplasma-positive sample. For insect pools, these combinations cannot be unequivocally assigned to a single phytoplasma genome.

## Data Availability

DNA sequences generated in the present research were deposited in the GenBank database under the following accession nos: PZ792957 to PZ792986 for *aceF*, PZ792987 to PZ793016 for *pnp*, PZ793017 to PZ793046 for *imp*, PZ793047 to PZ793076 for *secY*, and QB058825 to QB058826 for mitochondrial 16S rRNA genes.

## Supplementary Materials

The following supporting information can be downloaded at: https://www.mdpi.com/article/10.3390/plants15213432/s1, Figure S1: (**a**) and (**b**). Yellow sticky traps hung in the pear tree canopy at about 1.5 m from the ground with a high number of captured psyllids; Figure S2: Nested PCR amplification of a 16S rDNA fragment of pear decline (PD) phytoplasma occurring in southern Italy (examples) and reference phytoplasma strains of the apple proliferation (AP) phytoplasma group or 16SrX group using the universal phytoplasma primer pair P1/P7, followed by the group-specific primer pair fO1/rO1; Figure S3: (**a**) RsaI and (**b**) SspI restriction patterns of 16S rDNA amplified with the group-specific primer pair fO1/rO1 of pear decline (PD) phytoplasma occurring in southern Italy (examples) and reference phytoplasma strains of the apple proliferation (AP) phytoplasma group or 16SrX group; Table S1: Phytoplasmas related to pear decline (PD) phytoplasma occurring in southern Italy examined in this study; Table S2: Details of oligonucleotide primers used in this study; Supplementary Data: Individual gene alignments, concatenated partitioned alignment, and final tree in Newick format.
