# Supplementary material for: Occurrence, Epidemiological Aspects and Multilocus Sequence Typing of Pear Decline Phytoplasma in Southern Italy
Source: Plants (Basel). 2026 Nov 4;15(21):3432. doi: 10.3390/plants15213432 (PMC13765913; doi:10.3390/plants15213432)
Supplement: Supplementary file 1 [file plants-15-03432-s001.zip › Supplementary Figures-PD-new.pdf]

Supplementary Figures

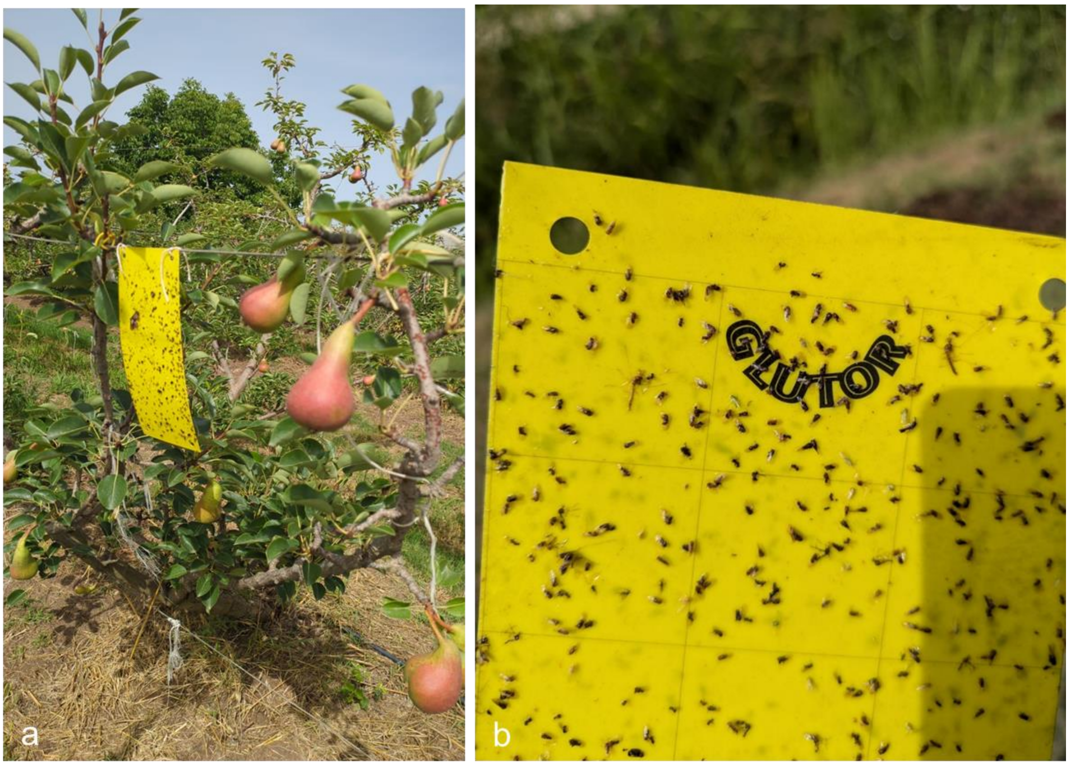

Figure S1. (**a**) and (**b**). Yellow sticky traps hung in the pear tree canopy at about 1.5 m from the ground with a high number of captured psyllids.

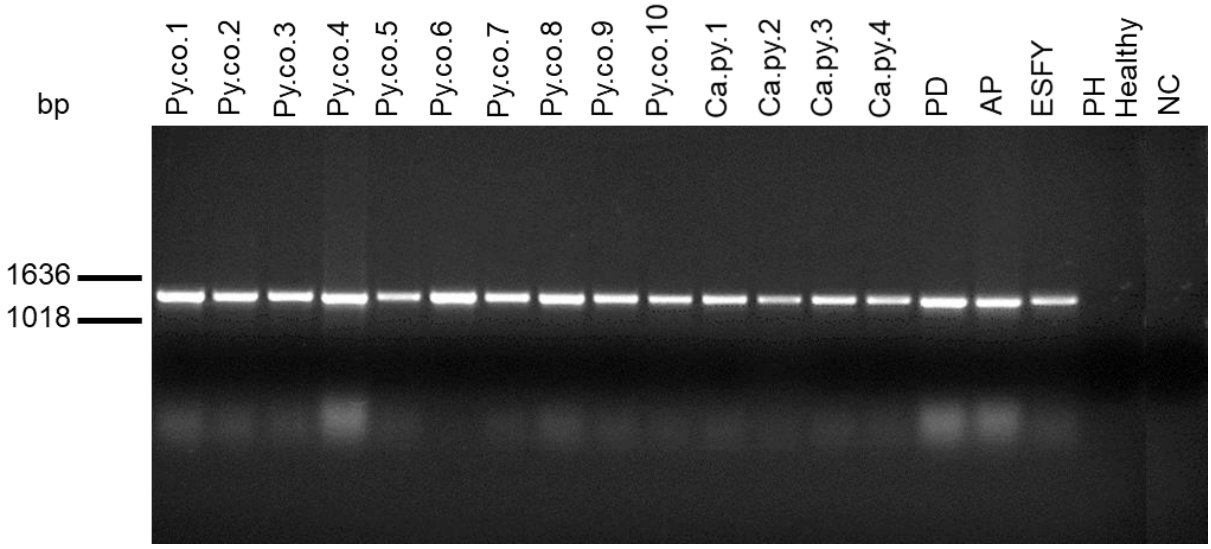

Figure S2. Nested PCR amplification of a 16S rDNA fragment of pear decline (PD) phytoplasma occurring in southern Italy (examples) and reference phytoplasma strains of the apple proliferation (AP) phytoplasma group or 16SrX group using the universal phytoplasma primer pair P1/P7 followed by the group-specific primer pair fO1/rO1. Py.co.1 to Py.co.10, samples from diseased pear trees; Ca.py.1 to Ca.py.4, samples from pear psylla *Cacopsylla pyri*, PD, pear decline (strain PD1); AP, apple proliferation (strain AT); ESFY, European stone fruit yellows (strain GSFY1); PH, nonsymptomatic pear; Healthy, healthy pear; NC, reaction mixture devoid of DNA template (negative control). The gel was photographed with the ImageQuant LAS 4000 system, version 1.2 (www.gelifesciences.com) and labelled with Photoshop CS3, version 10.0.1 (www.adobe.com).

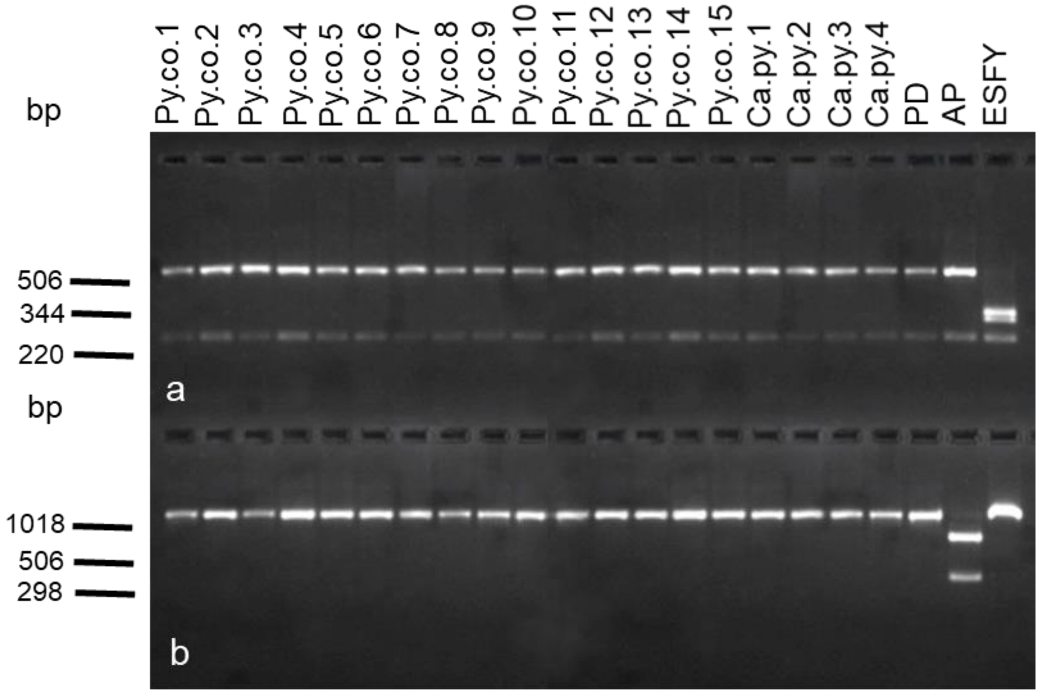

Figure S3. (**a**) *Rsa*I and (**b**) *Ssp*I restriction patterns of 16S rDNA amplified with the group-specific primer pair fO1/rO1 of pear decline (PD) phytoplasma occurring in southern Italy (examples) and reference phytoplasma strains of the apple proliferation (AP) phytoplasma group or 16SrX group. Py.co.1 to Py.co.15, samples from diseased pear trees; Ca.py.1 to Ca.py.4, samples from pear psylla *Cacopsylla pyri*, PD, pear decline (strain PD1); AP, apple proliferation (strain AT); ESFY, European stone fruit yellows (strain GSFY1). The gel was photographed with the ImageQuant LAS 4000 system, version 1.2 (www.gelifesciences.com) and labelled with Photoshop CS3, version 10.0.1 (www.adobe.com).
