# Supplementary material for: Occurrence, Epidemiological Aspects and Multilocus Sequence Typing of Pear Decline Phytoplasma in Southern Italy
Source: Plants (Basel). 2026 Nov 4;15(21):3432. doi: 10.3390/plants15213432 (PMC13765913; doi:10.3390/plants15213432)
Supplement: Supplementary file 1 [file plants-15-03432-s001.zip › Supplementary Tables-PD-new.pdf]

Supplementary Tables

Table S1. Phytoplasmas related to pear decline (PD) phytoplasma occurring in southern Italy examined in this study.

Phytoplasma (strain)	Original host	Geographical origin	Sequence	GenBank accession No.	Reference/collector(s)
Pear decline (PD)	*Pyrus communis*	Germany	*aceF*	FN598177	[18]
Pear decline (TR1)	*Pyrus communis*	Germany	*aceF*	FN598183	[18]
Pear decline (PD121Lib)	*Pyrus communis*	Lebanon	*aceF*	FN598178	[18]
Pear decline (PD2Luca)	*Pyrus communis*	Italy	*aceF*	FN598179	[18]
Pear decline (PHRZG1)	*Pyrus communis*	Croatia	*aceF*	FN598180	[18]
Pear decline (PIHRZG1)	*Cacopsylla pyrisuga*	Croatia	*aceF*	FN598181	[18]
Pear decline (PTK7)	*Cacopsylla pyri*	Turkey	*aceF*	FN598182	[18]
Pear decline (PDH16-20)	*Pyrus communis*	Switzerland	*aceF*	MG764529	[Toenshoff E.R., Brunner H., Oberhaensli T.]
Pear decline (THA-39)	*Pyrus communis*	Austria	*aceF*	MW456644	[49]
Pear decline (HH-1751)	*Cacopsylla pyricola*	Austria	*aceF*	MW456646	[49]
Pear decline (D877-07)	*Pyrus communis*	Slovenia	*aceF*	KF849465	[46]
Pear decline (D119-08)	*Pyrus communis*	Slovenia	*aceF*	KF849466	[46]
Pear decline (D685-08)	*Pyrus communis*	Slovenia	*aceF*	KF849467	[46]
Pear decline (D1040-07)	*Pyrus communis*	Slovenia	*aceF*	KF849468	[46]
Pear decline (D1041-07)	*Pyrus communis*	Slovenia	*aceF*	KF849469	[46]
European stone fruit yellows (WJ3703-30)	*Prunus armeniaca*	Germany	*aceF*	FN598166	[18]
European stone fruit yellows (ESFY)	*Prunus armeniaca*	Italy	*aceF*	FN598173	[18]
European stone fruit yellows (E60-11B)	*Cacopsylla pruni*	Italy	*aceF*	FN598176	[18]
European stone fruit yellows (C14)	*Cacopsylla pruni*	Czech Republic	*aceF*	PP176480	[72]
Apple proliferation (AP13)	*Malus domestica*	Romania	*aceF*	FN598185	[18]
Apple proliferation (AP032-10)	*Malus domestica*	France	*aceF*	FN598186	[18]
Pear decline (PD)	*Pyrus communis*	Germany	*pnp*	FN598192	[18]
Pear decline (PD121Lib)	*Pyrus communis*	Lebanon	*pnp*	FN598193	[18]
Pear decline (PD84)	*Pyrus communis*	Italy	*pnp*	FN598194	[18]
Pear decline (PD356-1)	*Pyrus communis*	France	*pnp*	FN598195	[18]
Pear decline (PD36AZ)	*Pyrus communis*	Azerbaijan	*pnp*	FN598196	[18]
Pear decline (PD2Luca)	*Pyrus communis*	Italy	*pnp*	FN598197	[18]
Pear decline (PHRZG1)	*Pyrus communis*	Croatia	*pnp*	FN598198	[18]
Pear decline (PIHRZG1)	*Cacopsylla pyrisuga*	Croatia	*pnp*	FN598199	[18]
European stone fruit yellows (WJ3703-30)	*Prunus armeniaca*	Germany	*pnp*	FN598190	[18]
European stone fruit yellows (C19)	*Cacopsylla pruni*	Czech Republic	*pnp*	PP176489	[72]
European stone fruit yellows (C7)	*Cacopsylla pruni*	Czech Republic	*pnp*	PP176490	[72]
Apple proliferation (AP28)	*Malus domestica*	Switzerland	*pnp*	FN598202	[18]
Pear decline (PD280-4)	*Pyrus communis*	France	*imp*	FN600721	[18]
Pear decline (PD20)	*Pyrus communis*	UK	*imp*	FN600722	[18]
Pear decline (PD36AZ)	*Pyrus communis*	Azerbaijan	*imp*	FN600723	[18]
Pear decline (PD121Lib)	*Pyrus communis*	Lebanon	*imp*	FN600724	[18]
Pear decline (PD33Lib)	*Pyrus communis*	Lebanon	*imp*	FN600725	[18]
Pear decline (PD)	*Pyrus communis*	Germany	*imp*	FN600726	[18]
Pear decline (PDSpa2)	*Pyrus communis*	Spain	*imp*	FN600727	[18]
Pear decline (PIHRKT1)	*Cacopsylla pyri*	Croatia	*imp*	FN600728	[18]
Pear decline (PIHRZG1)	*Cacopsylla pyrisuga*	Croatia	*imp*	FN600729	[18]
Pear decline (THA-38)	*Pyrus communis*	Austria	*imp*	MW456649	[49]
Pear decline (THA-39)	*Pyrus communis*	Austria	*imp*	MW456650	[49]
Pear decline (HH-284)	*Cacopsylla pyrisuga*	Austria	*imp*	MW456651	[49]
Pear decline (HH-1751)	*Cacopsylla pyricola*	Austria	*imp*	MW456652	[49]
Pear decline (HH-1766)	*Cacopsylla pyricola*	Austria	*imp*	MW456653	[49]
Pear decline (THA-14)	*Pyrus communis*	Austria	*imp*	MW456654	[49]
Pear decline (AA824)	*Pyrus communis*	Czech Republic	*imp*	MF374924	[47]
Pear decline (AA826)	*Pyrus communis*	Czech Republic	*imp*	MF374925	[47]
Pear decline (AA895)	*Pyrus communis*	Czech Republic	*imp*	MF374926	[47]
Pear decline (AA973)	*Pyrus communis*	Czech Republic	*imp*	MF374927	[47]
Pear decline (AB860)	*Pyrus communis*	Czech Republic	*imp*	MF374928	[47]

Peach yellow leaf roll (Mass1)	*Prunus persica*	Spain	*imp*	HG737344	[74]
Peach yellow leaf roll (PYLR)	*Prunus persica*	USA	*imp*	AF400589	[44]
European stone fruit yellows (ESFY)	*Prunus armeniaca*	Italy	*imp*	FN600707	[18]
Apple proliferation (AT)	*Malus domestica*	Germany	*imp*	FN600733	[18]
Pear decline (PDSpa2)	*Pyrus communis*	Spain	*secY*	FN598208	[18]
Pear decline (PD36AZ)	*Pyrus communis*	Azerbaijan	*secY*	FN598209	[18]
Pear decline (PD356-1)	*Pyrus communis*	France	*secY*	FN598210	[18]
Pear decline (PD2Luca)	*Pyrus communis*	Italy	*secY*	FN598212	[18]
Pear decline (LV72)	*Pyrus communis*	Italy	*secY*	MT321503	[Loreto G., Rizza S., Tessitori M.]
Pear decline (DSM)	*Pyrus communis*	Italy	*secY*	MT321504	[Loreto G., Rizza S., Tessitori M.]
Pear decline (PD1)	*Pyrus communis*	Italy	*secY*	GU004363	[75]
Pear decline (Po1)	*Pyrus communis*	Italy	*secY*	PX626289	[76]
Pear decline (Po2)	*Pyrus communis*	Italy	*secY*	PX626290	[76]
Pear decline (D55-08)	*Pyrus communis*	Slovenia	*secY*	KF849470	[46]
European stone fruit yellows (C19)	*Cacopsylla pruni*	Czech Republic	*secY*	PP176509	[72]
European stone fruit yellows (GSFY2)	*Prunus armeniaca*	Germany	*secY*	FN598205	[18]
Apple proliferation (AT)	*Malus domestica*	Germany	*secY*	FN598213	[18]

Table S2. Details of oligonucleotide primers used in this study.

Primer	Primer sequence (5'-3')	Sense	Target	References
P1	AGA-GTT-TGA-TCC-TGG-CTC-AGG-A	Forward	16S rDNA	[103]
P7	CGT-CCT-TCA-TCG-GCT-CTT	Reverse	23S rDNA	[103]
fO1	CGG-AAA-CTT-TTA-GTT-TCA-GT	Forward	16S rDNA	[83]
rO1	AAG-TGC-CCA-ACT-AAA-TGA-T	Reverse	16S rDNA	[83]
fPD	GAC-CCG-TAA-GGT-ATG-CTG-A	Forward	16S rDNA	[83]
AceFf1	TAA-AAT-TCG-CTG-ATG-TTG-GCG	Forward	*aceF*	[18]
AceFr1	CAT-CTT-TCA-ATT-CAT-TAA-AAC-TAG	Reverse	*aceF*	[18]
AceFf2	AGG-TAT-TGA-AGA-AGG-AAC-TG	Forward	*aceF*	[18]
AceFr2	CAA-CCG-CTT-TCA-TAA-TAA-AAG	Reverse	*aceF*	[18]
Pnpf1	GAA-GTT-GGT-ATT-ACT-GCT-TTA-C	Forward	*pnp*	[18]
Pnpr1	GAT-AAA-TCT-ATT-TGA-CCG-CG	Reverse	*pnp*	[18]
Pnpf2	TAC-AAT-TAG-ATA-TTA-AAG-TTA-AAG-G	Forward	*pnp*	[18]
Pnpr2	ATT-AAT-TTT-AAT-ACA-TTT-CGC	Reverse	*pnp*	[18]
IMPF2bis	GTA-TTT-AGT-TAC-AAT-TAT-TTA-CTA-G	Forward	*imp*	[18]
IMPR1bis	CAA-GAC-CTT-TAA-GGC-CAC-ATC	Reverse	*imp*	[18]
IMPF3pyr	AGG-AGA-AAA-AAT-AAT-GGA-AGC	Forward	*imp*	[18]
IMPR4pyrA	TTG-TGT-TCT-AAA-AGT-ATC-AC	Reverse	*imp*	[18]
SecYMalF1	TTA-GGA-CGT-AGT-ATA-CAA-ATC-CCN-TT	Forward	*secY*	[18]
SecYMalR1	ACA-ATA-ATT-AAA-AAT-CCT-GTN-CC	Reverse	*secY*	[18]
SecYMalF2	AAG-AAT-GGC-GTG-AAC-ARG-GNG-A	Forward	*secY*	[18]
SecYMalR2	GCA-TCT-TGT-TTA-GAT-AAA-TGT-TC	Reverse	*secY*	[18]
16SR21	GCG-TGT-TTA-TCA-AAA-ACA-T	Forward	mitochondrial 16S rDNA	[104]
16S22	CCG-GTC-TGA-ACT-CAG-ATC-A	Reverse	mitochondrial 16S rDNA	[104]
